# Lighting-Up the Far-Red Fluorescence of RNA-Selective Dyes by Switching from Ortho to Para Position

**DOI:** 10.3390/ijms24054812

**Published:** 2023-03-02

**Authors:** Alessio Cesaretti, Eleonora Calzoni, Nicolò Montegiove, Tommaso Bianconi, Martina Alebardi, Maria Antonietta La Serra, Giuseppe Consiglio, Cosimo Gianluca Fortuna, Fausto Elisei, Anna Spalletti

**Affiliations:** 1 Department of Chemistry, Biology and Biotechnology and Center of Excellence on Innovative Nanostructured Materials (CEMIN), University of Perugia, via Elce di Sotto 8, 06123 Perugia, Italy; 2 Department of Chemical Sciences, University of Catania, Viale Andrea Doria 6, 95125 Catania, Italy

**Keywords:** fluorescent probe, push-pull dye, intramolecular charge transfer, RNA-selectivity, deep-red emission, fluorescence microscopy, nucleolar RNA, mitochondrial RNA, bioimaging, theranostics

## Abstract

Fluorescence imaging is constantly searching for new far-red emitting probes whose turn-on response is selective upon the interaction with specific biological targets. Cationic push-pull dyes could indeed respond to these requirements due to their intramolecular charge transfer (ICT) character, by which their optical properties can be tuned, and their ability to interact strongly with nucleic acids. Starting from the intriguing results recently achieved with some push-pull dimethylamino-phenyl dyes, two isomers obtained by switching the cationic electron acceptor head (either a methylpyridinium or a methylquinolinium) from the ortho to the para position have been scrutinized for their ICT dynamics, their affinity towards DNA and RNA, and in vitro behavior. By exploiting the marked fluorescence enhancement observed upon complexation with polynucleotides, fluorimetric titrations were employed to evaluate the dyes’ ability as efficient DNA/RNA binders. The studied compounds exhibited in vitro RNA-selectivity by localizing in the RNA-rich nucleoli and within the mitochondria, as demonstrated by fluorescence microscopy. The para-quinolinium derivative showed some modest antiproliferative effect on two tumor cell lines as well as improved properties as an RNA-selective far-red probe in terms of both turn-on response (100-fold fluorescence enhancement) and localized staining ability, attracting interest as a potential theranostic agent.

## 1. Introduction

Nucleic acids fully deserve the epithet of molecules of life. All genetic information residing in the DNA sequences is transferred from DNA to the RNA molecule for its translation into proteins which carry out vital functions in the organisms. The possibility of following nucleic acids inside a cell has always attracted vivid interest, and the use of fluorescence imaging is the natural answer to this demand [1]. The number of fluorescent probes able to interact with nucleic acids is ever-increasing, and research is constantly looking at the identification of novel small molecules which possess the finest properties for their fluorescence response to be aptly tuned [2,3,4].

As a rule, a staining dye to be used in bioimaging has to exhibit some essential characteristics [5,6]: (a) the ability to cross cell membranes, which is granted by a specific balance between hydrophilicity, for stability in an aqueous environment, and lipophilicity, for easy passage through phospholipid bilayers; (b) high binding affinity for their biological targets; (c) emission turn-on upon complexation with biomolecules inside the cell to visualize specific organelles and reduce background fluorescence from free dye molecules; (d) large Stokes shifts to avoid self-quenching and fluorescence in the deep-red to ensure better tissue penetration and avoid both photo-damage and the contribution of autofluorescence from the surrounding environment.

Some structural motifs have proved effective in boosting the affinity for nucleic acids: aromatic portions can favor interactions with nucleobases, while positive charges allow electrostatic attraction with the negatively charged phosphate backbone [7,8,9,10]. This may result in either intercalation of the probe between adjacent nucleobases or binding in the major and minor grooves of the nucleic acids [11,12,13,14]. These structural properties can be combined in molecules endowed with push-pull character, where electron acceptor (A) and electron donator (D) portions are linked by a π-conjugated bridge in a basic A-π-D arrangement or more articulate A-π-D-π-A and D-π-A-π-D structures. This allows, on the one hand, shifting the optical properties to the visible up to the far-red by increasing conjugation and, on the other hand, fostering a turn-on behavior. In fact, push-pull molecules undergo intramolecular charge transfer (ICT) transitions upon photoexcitation, with their excited state being stabilized in a polar environment, where its deactivation to the ground state takes place mainly by internal conversion. Under these conditions, to favor the charge separation and the following stabilization, a push-pull A-π-D molecule can undergo a 90-degree rotation around the π-bridge leading to a nearly non-fluorescent twisted ICT (TICT) state [15,16,17]. When these dyes are instead confined in a rigid environment, like that provided by a nucleic acid or an increase in viscosity, the restriction of molecular motion prevents the ICT state from reaching a fully-relaxed TICT state with a consequent enhancement of its fluorescence [16,18,19].

However, selectivity, the capability of a molecule to stain particular organelles or interact preferentially with a specific biological target over others, is another highly required feature a good probe should exhibit [20,21,22,23]. When it comes to RNA, selectivity allows RNA domains to be visualized either inside the cell nuclei, within the nucleoli, or in the cytoplasm, where it amasses in specific organelles such as mitochondria and ribosomes [24,25]. The interest in RNA imaging derives from the implication of this nucleic acid in a number of cell processes, which include, besides common protein synthesis, post-transcriptional and gene expression regulations being fundamental in the cell vital cycle [26]. Further to this, RNA is involved in the replication of so-called RNA viruses, which represent a very current healthcare issue [16]. The use of selective fluorescence probes specifically interacting with RNA could thus become a powerful tool not only to perform imaging for localization and diagnostic purposes but also to monitor and interfere with cell viability or virus replication for the treatment of diseases, whether tumors or infections, respectively [5].

As of today, SYTO^TM^ RNASelect^TM^ is one of the few fluorescent dyes available in the market for RNA staining [25]. It exhibits a bright fluorescence in the green upon binding to RNA, as opposed to a weak fluorescence for its DNA-bound form, allowing RNA to be visualized in nucleoli and cytoplasm. A lot of research study has been running rampant to look for other small molecules that could be used as an alternative to SYTO^TM^ RNASelect^TM^, with the deliberate aim to outdo its performances in terms of selectivity and emission properties [27,28,29,30,31].

**Scheme 1:**
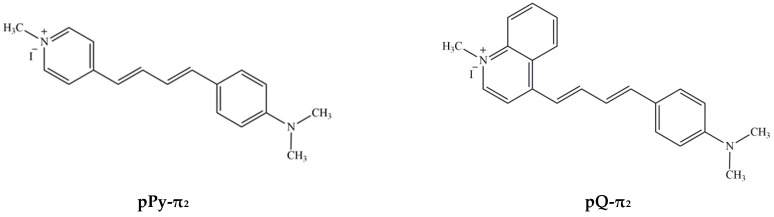
Molecular structures of the two investigated compounds.

In our long-term research concerning the interaction between small organic molecules and biological targets [32,33,34,35], we have recently come across some push-pull styryl compounds which indeed show certain RNA selectivity [5,36]. In particular, a recent work of ours [36] deals with a series of three A^+^-π-D dyes, where D is a dimethylamino-phenyl group, A^+^ is either a methyl pyridinium or methyl quinolinium, and the π-bridge is a simple ethylene or extended butadiene. These probes, studied in a tumor cell line by confocal fluorescence, were found to mostly stain nucleoli as a consequence of the interaction with RNA, as proven by the ribonuclease A (RNase) digest test. Further to this, in another study [5], we scrutinized a series of A^+^-π-D-π-A^+^ dyes and found that a greater affinity for RNA over DNA was exhibited for the compound with an electron donor methyl quinolinium attached in the para position relative to the π-bridge. On the basis of the previous findings, two push-pull dyes (shown in Scheme 1) were designed. They are the analogs of compounds already studied in ref. [36] and obtained by switching from ortho to para position the A^+^ moiety in the A^+^-π-D dimethylamino-phenyl, and investigated for their application as deep-red RNA-selective fluorescent probes. By doing so, the turn-on response guaranteed by the strong electron donor dimethylamino-phenyl was combined within the same molecular structures with the selectivity granted by the attachment in the para position.

As a matter of fact, while developing this project, we came across a recent work by Zhang et al. [37] where one of the two para isomers here presented (i.e.*,* the quinolinium derivative, **pQ-π_2_**) had already been tested as a far-red fluorescent probe both in vitro and in vivo. Its turn-on response was interpreted as due to the increased viscosity sensed within the cells, but the possible interaction with nucleic acids, which represent an obvious biological target for these positively-charged aromatic small molecules, was completely overlooked and left out of the discussion.

In this work, a thorough spectroscopic investigation was carried out for the two para isomers as a function of solvent polarity, allowing the push-pull character of the two dyes to be described in terms of dipole moment variation upon excitation (Δµ) and hyperpolarizability coefficients (β), as derived from their solvatochromic behavior; the role of photoinduced ICT in the excited-state dynamics of the two compounds was also deduced from careful analyses of ultrafast transient absorption and fluorescence up-conversion measurements. In addition, their binding affinities with nucleic acids as well as the photophysical properties of the free dyes compared to their bound forms, were evaluated *ex cellula* by spectrophotometric and fluorimetric titrations (with both tRNA and ct-DNA) and femtosecond-resolved spectroscopies; while the effective selectivity towards RNA was assessed in vitro through fluorescence microscopy. In particular, the two dyes were found to localize in the nucleoli and the mitochondria, driven by the explicit interaction with RNA, with better performances revealed for the quinolinium derivative.

## 2. Results

### 2.1. Photophysical Properties

The two investigated compounds were first studied for their photophysical behavior to evaluate the entity of their push-pull character. The spectral properties of **pPy-π_2_** had already been analyzed in previous work [38], while **pQ-π_2_** was here studied in a wide range of solvent polarities for the first time.

**Figure 1:**
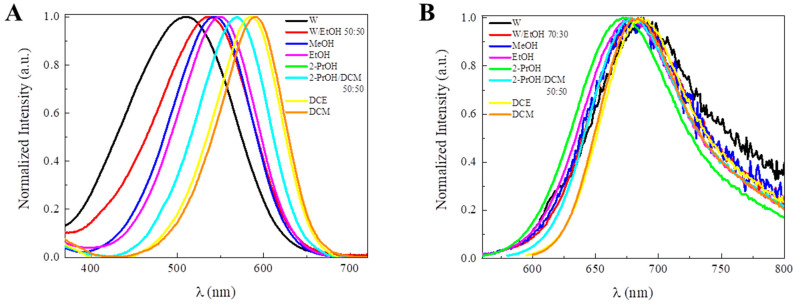
Absorption (**A**) and emission (**B**) spectra of compound **pQ-π_2_** in solvents of different polarity. (W—water; EtOH—ethanol; MeOH—methanol; 2-PrOH—2-propanol; DCE—1,2-dichloroethane; DCM—dichloromethane).

A marked negative solvatochromic behavior was observed for the absorption spectrum of both dyes (Table 1 and Figure 1 and Figure S1), whose maximum shifts to higher energy as the polarity increases as a result of the greater stabilization of the more polar ground state relative to the excited state reached upon excitation. The emission spectrum is instead almost insensitive to the polarity of the medium, as expected for a relaxed excited state with an ICT nature [38], but when it comes to **pQ-π_2_**, its emission is somewhat affected by viscosity, which causes a modest enlargement of the spectrum on the blue side of the band (Figure 1B), supposedly because of the contribution of different excited-state configurations.

On the basis of Reichardt theory [39], solvatochromism was used to derive the difference in dipole moments between the ground and the Franck-Condon excited state (Δμ_exp_) from the slope of the linear fitting obtained by plotting the Stokes shift vs. the $E_{T}^{N}$ parameter, which accounts for the polarity of the solvent (Figure S2). The Δμ_exp_ absolute values proved to be higher than 10 D for both compounds (Table S1). This analysis also allowed the first hyperpolarizability coefficient (β_0_), which is an indicator of the push-pull character of the molecule, to be deduced through the solvatochromic method, as reported in Table S1 [38,40]. The highest values (Δμ_exp_ = −13.2 D and β_0_ = 200 × 10^−30^ esu^−1^ cm^5^) were found for **pPy-π_2_**, likely because it features a highly conjugated linear structure which extends along the direction of the charge transfer from the dimethyl-phenyl group to the methyl pyridinium, while the additional ring in **pQ-π_2_** is placed outside of the CT direction.

In fact, quantum mechanical calculations, previously performed on **pPy-π_2_** [38] and here carried out for **pQ-π_2_** at the same TD-DFT level of theory to optimize the ground state geometry and describe the lowest singlet excited states, revealed a greater effect on the electron density displacement upon absorption in the case of **pPy-π_2_** (Figures S3–S6 and Tables S2 and S3).

**Figure 2:**
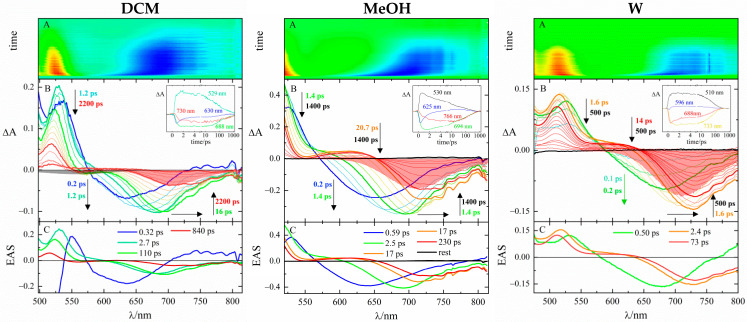
Femtosecond transient absorption (fs-TA) measurements of compound **pPy-π_2_** in DCM (**left**), MeOH (**middle**), and W (**right**) obtained by pump-probe experiment (λ_exc_ = 400 nm): panel A, experimental 3D matrix reporting color-coded ΔA as a function of wavelength and time (ΔA > 0 yellow-orange, ΔA < 0 blue); panel B, representative spectra at different delay times and representative kinetics (inset) at different wavelengths, together with steady-state absorption (gray-shaded area) and fluorescence (red-shaded area); panel C, EAS (evolution-associated spectra) obtained by Global Analysis.

The ICT nature of the relaxed excited state was instead experimentally evidenced by the quenching of the fluorescence quantum yields (Table 1) with increasing solvent polarity by more than one order of magnitude when going from a sparingly polar solvent, i.e., DCM, to an aqueous environment (Φ_F,DCM_/Φ_F,water_ = 45 and 26 for **pPy-π_2_** and **pQ-π_2_**, respectively). The conclusive proof, however, was provided by the excited state dynamics investigated by fs-transient absorption (fs-TA) and fs-fluorescence up-conversion (fs-FUC). The former was performed in DCM, MeOH, and W to assess the effect of polarity (Figure 2 and Figure S7 and Table 2). Although fs-TA experiments had already been carried out in the case of **pPy-π_2_** [38], its measurements were repeated for this work. The new data fairly reproduced the published dynamics, but their thorough analysis, assisted by the comparison with the new fs-FUC measurements, led to a slightly modified interpretation. The time-resolved transient absorption spectra are characterized by positive excited-state absorption signals and negative bands due to either ground-state bleaching (GSB), matching the steady-state absorption region, or stimulated emission (SE). The evolution of the SE band, sensitive to stabilization of the excited state, reveals important dynamics at short delays in all the media analyzed, suggesting the presence of some ICT process, with SE overlapping the steady-state fluorescence only at longer times. In fact, the time associated with the longer transient, corresponding to the lifetime of the S_1_ state, was found to greatly reduce as the polarity increases (τ_S1,DCM_ = 840 ps and τ_S1,w_ = 73 ps for **pPy-π_2_** and τ_S1,DCM_ = 82 ps and τ_S1,w_ = 5.6 ps for **pQ-π_2_**) as it is typical of ICT states mainly decaying by internal conversion in a polar environment. In particular, shorter times are peculiar to **pQ-π_2_** supposedly because of a better ability to separate the charge in the relaxed ICT state.

**Figure 3:**
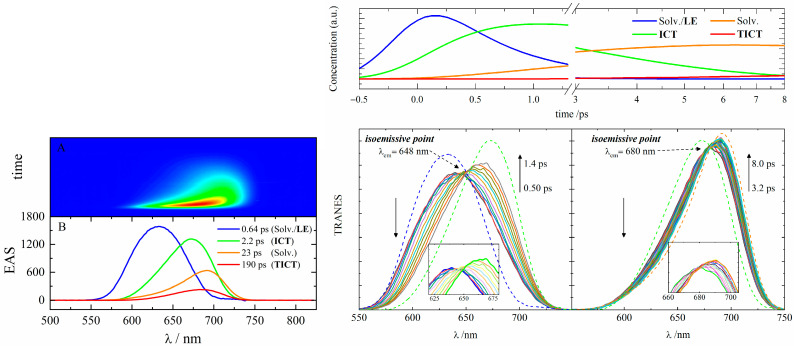
Femtosecond fluorescence up-conversion (fs-FUC) measurement of compound **pPy-π_2_** in MeOH obtained by pump-probe experiment (λ_exc_ = 400 nm, **left**): panel **A**, experimental 3D matrix reporting color-coded ΔA as a function of wavelength and time (ΔA = 0 blue, ΔA > 0 increasing from green to red); panel **B**, EAS obtained by Global Analysis. Time-resolved area-normalized emission spectra (TRANES) analysis (**right**): upper panel, concentration profiles for the transient species detected by Global Analysis; lower panels, TRANES evolution over time calculated in proper delay time intervals together with the EAS obtained by Global Analysis (dashed lines) reported as limit spectra.

Femtosecond fluorescence up-conversion (fs-FUC) measurements, carried out in polar MeOH, disclosed the ICT dynamics of the two investigated push-pull dyes (Figure 3 and Figure S8). The time-resolved emission spectra follow the stabilization of the emissive excited state with time (Figure 3 and S8, left), which could be related to either solvation or the population of different states. In the case of **pPy-π_2_**, the Global Analysis returned four transients (in line with the results of fs-TA, cf. Table 2), three matching the canonical solvation times of MeOH (τ = 0.64 ps, 2.2 ps, and 23 ps) [41] and the longest one τ_S1_ = 190 ps assigned to the relaxed emissive state. The time-resolved emission spectra were then processed to give TRANES (time-resolved area-normalized emission spectra) [18,42], which revealed the existence of a three-state dynamic masked by solvation, as evidenced by the presence of two distinct isoemissive points in their evolution (Figure 3, right). In analogy with what has previously been observed for other push-pull methylpyrydinium derivatives [18], the dynamics can be interpreted as an ultrafast charge transfer process from the locally-excited (LE) state, happening together with inertial solvation (τ_LE→ICT_ = 0.64 ps), and a rotation around the π-bridge that favors the charge separation to form a twisted ICT (TICT) state (τ_ICT→TICT_ = 2.2 ps) during diffusive solvation, which later undergoes a second diffusive solvation step (τ_solv._ = 23 ps) before returning to the ground state with a lifetime of 190 ps. The twisted geometry of the TICT state is also corroborated by a reduction of the full width at half maximum during the spectral evolution, as it is common for an emissive state whose population distribution narrows around the equilibrium twisted position [43]. These dynamics account for both the large bathochromic shift of the emission up to the far-red region (ICT character) and the scarce fluorescence in polar media (twisted geometry). As for **pQ-π_2_** (Figure S8), because of its faster dynamics and owing to the reduced temporal resolution of the fs-FUC setup, the first transient ascribable to the LE state (τ_LE_ = 0.46 ps from the fs-TA experiment) could not be detected, and therefore the TRANES evolution only revealed the transition from the ICT state to the TICT state (τ_ICT→TICT_ = 1.5 ps), followed by diffusive solvation (τ_solv._ = 4.9 ps) and excited-state deactivation (τ_TICT→S0_ = 15 ps).

### 2.2. Interaction with Nucleic Acids

The absorption spectra of the two molecules in buffered water at pH 7.4, mimicking a biological environment, feature a broad band centered at 447 and 511 nm for **pPy-π_2_** and **pQ-π_2_**, respectively, with a trend that parallels the increased conjugation given by the substitution of the pyridine rings with a quinolinium. The emission is instead observed in the deep red with maxima at 709 and 688 nm, resulting in very large Stokes shifts of 8270 and 5000 cm^−1^ for **pPy-π_2_** and **pQ-π_2_**, respectively. The fluorescence quantum yields are very low, with the quinolinium derivative (Φ_F_ = 0.002) being less fluorescent than the pyridinium one (Φ_F_ = 0.056).

**Figure 4:**
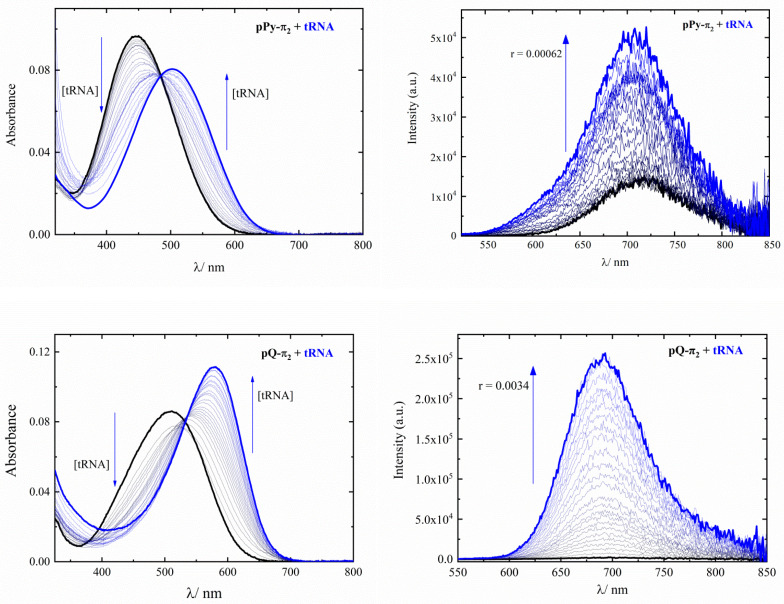
Changes in the absorption (**left panels**) and fluorescence (**right panels**) spectra of **pPy-π_2_** and **pQ-π_2_** in ETN buffer solution, pH 7.4, upon addition of increasing amounts of tRNA, with r = [compound]/[nucleic acid]. [**pPy-π_2_**] = 1.7 µM, λ_exc_ = 447 nm; [**pQ-π_2_]** = 1.5 µM, λ_exc_ = 510 nm.

The affinity for nucleic acids was evaluated through spectrophotometric and fluorimetric titration by adding increasing amounts of calf thymus DNA (ct-DNA) and tRNA up to a ratio *r* = [dye]/[nucleic acid] < 0.005, as shown in Figure 4 and Figure S9. The interaction of both compounds with either ct-DNA or tRNA caused the absorption spectra to undergo significant redshifts as a consequence of the exclusion of water molecules from the vicinity of the probe upon complexation. In the case of **pPy-π_2_**, the spectrophotometric titrations reveal a clear isosbestic point implying the existence of one predominant mode of binding, which is likely to be intercalation owing to the net hypochromicity detected for the absorption band [9]. As for **pQ-π_2_**, the spectral evolution showed deviations from the isosbestic point: as the concentration of nucleic acids increases, a first hypochromic effect is recorded, followed by an important enhancement of the absorbance (hyperchromic effect), which could instead be related to interaction by different modes of binding, possibly within the grooves of the polynucleotide chains. When it comes to spectrofluorimetric titrations, both dyes were found to light up by enhancing their emissive capability: **pPy-π_2_** showed a more than 10-fold increase in quantum efficiency (QE) with both nucleic acids (QE_F,ct-DNA_ = 0.084 and QE_F,tRNA_ = 0.073), while **pQ-π_2_** experienced a greater turn-on response, with a 42-fold fluorescent enhancement when bound to ct-DNA (QE_F,ct-DNA_ = 0.084) and an almost 100-fold increase after complexation with tRNA (QE_F,tRNA_ = 0.18). The huge change in the emission intensity allowed the processing of the titration data by the Scatchard equation to obtain the binding constants reported in Table 3 (Figure S10). As a rule, **pQ-π_2_** showed greater affinity (by almost one order of magnitude) as opposed to the pyridinium derivative, likely because of the more extended aromaticity.

**Figure 5:**
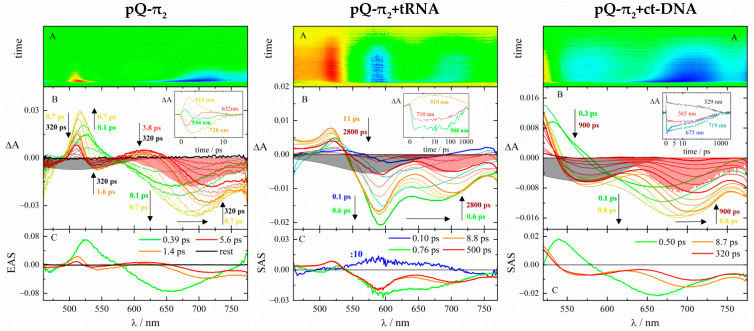
Femtosecond transient absorption (fs-TA) measurements of compound **pQ-π_2_** in ETN buffer solution, pH 7.4, alone (**left**) and in the presence of tRNA (**middle**) and ct-DNA (**right**), with r ([compound]/[nucleic acid]) = 0.02, obtained by pump-probe experiment (λ_exc_ = 400 nm): panel **A**, experimental 3D matrix reporting color-coded ΔA as a function of wavelength and time (ΔA > 0 yellow-orange, ΔA < 0 blue); panel **B**, representative spectra at different delay times and representative kinetics (inset) at different wavelengths, together with steady-state absorption (gray-shaded area) and fluorescence (red-shaded area); panel **C**, EAS (evolution-associated spectra) obtained by Target Analysis.

The dye/nucleic acid complexes were also characterized by the fs-TA measurements reported in Figure 5 for **pQ-π_2_** and in Figure S11 for **pPy-π_2_**. The signals recorded for the two dyes in an aqueous buffer at pH 7.4 in the presence of nucleic acid (*r* = 0.02) showed signs of the interaction by virtue of the red-shift of the GSB band, in line with the spectral changes observed during the spectrophotometric titrations, and the significant lengthening of the overall excited-state dynamics, which parallels the fluorescence enhancement achieved upon binding. The Target Analysis, whose results are listed in Table 4, confirmed these observations, always revealing a transient with a lifetime far exceeding that of the free dyes in water. The shortest components are assigned to solvation processes, but in the case of **pPy-π_2,_** a species characterized by a lifetime and a spectral profile resembling those of the free molecule in an aqueous solution can also be recognized. Such species are not present in the excited-state deactivation of **pQ-π_2_** under these conditions, thus validating the higher binding affinity assessed for the quinolinium derivative. More interestingly, **pQ-π_2_** features a longer lifetime when bound to tRNA (τ**_pQ-π2+tRNA_** = 500 ps) compared to its bound form with ct-DNA (τ**_pQ-π2+ct-DNA_** = 320 ps), in a parallel trend with the fluorescence enhancement. The fluorescence quantum yields and excited-state lifetimes allowed the fluorescence rate constants to be determined. They were found to be 0.77 × 10^8^ s^−1^ and 3.6 × 10^8^ s^−1^ in the buffer for **pPy-π_2_** and **pQ-π_2_**, respectively, and they did not change appreciably upon complexation, meaning that the ICT character of the emissive state is not altered by the interaction; hence, the lighting-up of the fluorescence response is mainly due to the protection-action played by the nucleic acids which shield the probes from the bulk aqueous environment, thus hindering their twisting, but still allowing a far-red emitting ICT state to be reached.

### 2.3. In Vitro Studies: Antiproliferative Effect and Intracellular Localization

The possibility of using the two dyes for staining purposes is subject to their ability to pass the cellular outer membrane, which was proved by treating human tumor cells in vitro (A549 and HT-29) with micromolar concentrations of the two molecules. The dyes, featuring the right balance between lipophilic and hydrophilic portions, were efficiently and rapidly internalized by the cells. The effect of the two compounds on cell viability was then evaluated by the MTT test performed after 24 h of incubation (Figure S12). Both molecules proved to be tolerated by the cells at concentrations ≤ 1 µM, while they exerted some antiproliferative effect at higher concentrations, especially toward HT-29 cells. In particular, **pQ-π_2_** exhibited moderate cytotoxicity, with an IC_50_ (IC_50_ = dye concentration inducing 50% inhibition of cell growth) of about 90 and 60 µM for A549 and HT-29 cells, respectively. As for **pPy-π_2_**, its IC_50_ against both cell lines was larger than 100 µM, and it can thus be regarded as noncytotoxic.

**Figure 6:**
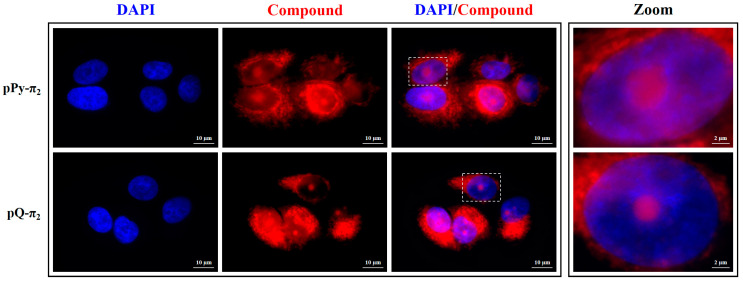
**Left**: representative fluorescence microscopy images of fixed A549 cells stained with DAPI (blue, DAPI filter), 10 µM **pPy-π_2_** and **pQ-π_2_** compounds (red, TRITC filter), and the relative merged images. Image magnification: 60×. **Right**: zoom-in of representative cells.

**Figure 7:**
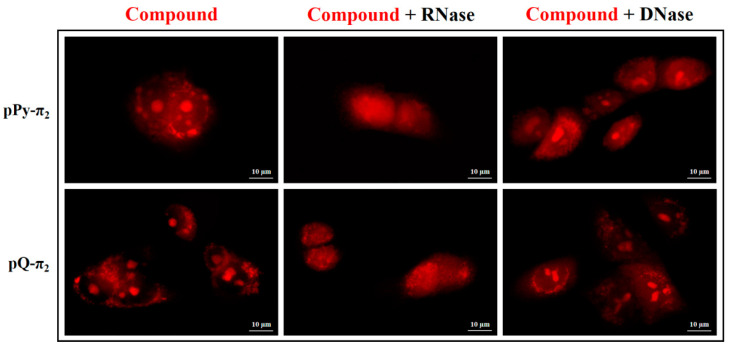
Representative fluorescence microscopy images of fixed and permeabilized A549 cells stained with 10 µM **pPy-π_2_** and **pQ-π_2_** compounds (red, TRITC filter) before and after treatment with RNase and DNase. Image magnification: 60×.

To understand the reasons for such an antiproliferative effect and assess the possibility of resorting to the two dyes as effective staining agents, their peculiar localization within A549 cells was investigated by fluorescence microscopy. After fixing the cells after 2 h of incubation, the bright and red emission of the two compounds was found not to be randomly diffused inside the cellular environment but specifically localized in certain organelles (Figure 6 and Figure 7). In particular, the perinuclear portion of the cytoplasm was lighted up, as well as some punctuate structures within the nuclei, which appeared particularly bright in the case **pQ-π_2_**. By co-staining the cells with the nuclear blue dye DAPI (Figure 6), the red dots in the nucleus, characterized by a signal which is spatially complementary to the blue fluorescence of DAPI, can be recognized as the nucleoli, proving the two investigated molecules to be permeant to the two lipid bilayers constituting the nuclear membrane. This finding envisages a certain RNA-selectivity for both compounds, inasmuch as nucleoli are rich in RNA, particularly ribosomal RNA (rRNA), while DNA has a three-fold higher concentration in the remaining nucleoplasm, being expressly illuminated by DAPI [12].

To validate the RNA selectivity of the two molecules and their localization in the nucleoli, ribonuclease (RNase) and deoxyribonuclease (DNase) digestion experiments were carried out on fixed and permeabilized A549 cells. Figure 7 reports representative fluorescence microscopy images of cells stained with the two probes before and after treatment with RNase and DNase. In the first case, as a consequence of the degradation of RNA, the emission loses its characteristic confinement in punctuate structures, while the fluorescence signal is practically not affected by the digestion of DNA following the action of DNase. This finding confirms that the punctuated bright emission comes from a specific interaction with the RNA within the nucleoli.

**Figure 8:**
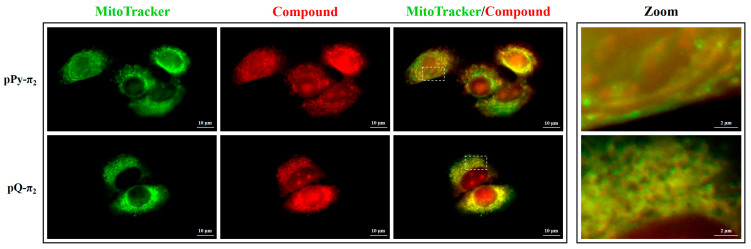
**Left**: representative fluorescence microscopy images of fixed A549 cells stained with 200 nM MitoTracker^TM^ (green, FITC filter), 10 µM **pPy-π_2_** and **pQ-π_2_** compounds (red, TRITC filter), and the relative merged images. Image magnification: 60×. **Right**: zoom-in of representative cells.

Moreover, in order to understand the origin of the red emission in the perinuclear region, colocalization experiments were carried out with a commercial dye specific for mitochondria staining, namely MitoTracker™ Green FM Dye. By virtue of the anticipated RNA selectivity, the two styryl dyes were expected to light up cellular compartments particularly rich in RNA, and mitochondria could indeed be a target for the investigated molecules, also because quaternary ammonium is a recurring motif in mitochondria probes [44,45]. The analysis of the fluorescence microscopy images, merged into yellow as reported in Figure 8, gave Pearson’s coefficients (Rr, describing the spatial colocalization) of 0.67 and 0.73 for **pPy-π_2_** and **pQ-π_2_**, respectively, implying moderate colocalizations [46,47,48]. In fact, the red emission of the dyes and the green emission of MitoTracker™ do not overlap in the nucleolar region, where only the push-pull dimethylamino-phenyl dyes can be spotted. This is supported by the computation of Manders’ coefficients (m_1_ and m_2_) for red-green colocalization, which indicates the fraction of red pixels overlapping with green (m_1_) and vice versa (m_2_) [47,49]. By doing so, a strong degree of colocalization can be found for the green (m_2_ = 0.98 and 0.99 for **pPy-π_2_** and **pQ-π_2_**, respectively), while the presence of the red fluorescence inside the nuclei is discriminated by the reduced m_1_ coefficient, which is 0.78 for **pPy-π_2_** and 0.90 **pQ-π_2_.** However, if only the perinuclear region is considered for the analysis of the images, the overlapping is strong for **pPy-π_2_** (with Rr = 0.88, m_1_ = 0.800, and m_2_ = 0.994) and almost complete for **pQ-π_2_** (with Rr = 0.94, m_1_ = 0.986, and m_2_ = 0.996).

## 3. Discussion

Two push-pull A^+^-π-D dyes, where D is a dimethylamino-phenyl group and A^+^ is either a methyl pyridinium or methyl quinolinium attached in the para position relative to the butadiene π-bridge, were investigated as far-red fluorescent probes to be compared to their ortho positional isomers, which had previously been studied in our laboratory [36]. Switching from the ortho to the para position causes the absorption spectrum recorded in water to shift bathochromically by about 10 nm, likely because of the more conjugative position of attachment. As for the fluorescence emission, it is always found in the deep-red region with broad bands centered around 700 nm. This results in huge Stokes shifts, larger in the case of the pyridinium derivatives (greater than 8000 cm^−1^), which is desirable for bioimaging applications. The fluorescence quantum yields are instead small (<1%) and not affected by the attachment position for the pyridinium derivatives (Φ_F_ = 0.0056 and 0.0064 for **pPy-π_2_** and **oPy-π_2_**, respectively); however, when it comes to the quinolinium-substituted molecules, the para position allows a five-fold increase in the Φ_F_ value (Φ_F_ = 0.0020 and 0.0004 for **pQ-π_2_** and **oQ-π_2_**, respectively). The large Stokes shift and reduced fluorescence were explained by invoking important excited-state dynamics with the population of twisted ICT states mainly decaying by non-radiative internal conversion, as proven by fs-TA and fs-FUC measurements. These dynamics are favored by the presence of the strong electron-donor moiety dimethylamino phenyl, implying a great push-pull character for these dyes. The push-pull nature of the investigated compounds was quantified by the computation of their first hyperpolarizability coefficients (β_0_) deduced from the straightforward negative solvatochromic behavior. The β_0_ values were found to enhance by switching from ortho to para position for the attachment of the pyridinium ring (β_0_ = 120 × 10^−30^ esu^−1^ cm^5^ for **oPy-π_2_ vs.** 200 × 10^−30^ esu^−1^ cm^5^ for **pPy-π_2_**) [38], but not to change upon position changes in the case of the quinolinium derivatives (β_0_ = 160×10^−30^ esu^−1^ cm^5^ for both **oQ-π_2_** and **pQ-π_2_**), supposedly because the additional aromatic ring in **pQ-π_2_** is not placed in the direction of the CT vector.

These photophysical properties (ICT dynamics, large Stokes shifts, far-red emissions) are alluring for a fluorescent probe as long as they are combined with a high affinity for biological targets. Spectrophotometric and fluorimetric titrations with ct-DNA and tRNA revealed the favorable interactions of the dyes with nucleic acids. In particular, **pQ-π_2_** exhibited one-order of magnitude higher associations constants than **pPy-π_2_** (K_ass,tRNA_ = 2.4 × 10^4^ M^−1^ vs. K_ass,tRNA_ = 4 × 10^3^ M^−1^, and K_ass,ct-DNA_ = 1.8×10^5^ M^−1^ vs. K_ass,ct-DNA_ = 3.0 × 10^4^ M^−1^), probably by virtue of the increased condensed aromatic surface. The greater affinity of **pQ-π_2_** also implies more pronounced changes in both the absorption spectrum (marked red-shift and hyperchromic effect) and the emission spectrum (sharp fluorescence enhancement). Moreover, by comparing these results with those already published for the ortho isomers with tRNA (K_ass,tRNA_ = 1.6 × 10^3^ M^−1^ for **oPy-π_2_** and K_ass,tRNA_ = 7 × 10^3^ M^−1^ for **oQ-π_2_**) [36], a peculiar greater affinity can be identified in the only case of **pQ-π_2_**. This finding is also accompanied by the highest emission turn-on response (almost 100-fold fluorescence enhancement) shown by the para isomer of the quinolinium derivative when bound to RNA, reaching a high quantum efficiency (QE) of 0.18, as opposed to the other compounds of the series (QE**_pPy-π2_**_+RNA_ = 0.073, QE**_oPy-π2_**_+RNA_ = 0.025, and QE**_oQ-π2_**_+RNA_ = 0.073) and competitive with the fluorescence ability of other RNA fluorescent probes [16,24]. In addition, unlike **pPy-π_2_**, the fs-TA analysis of the **pQ-π_2_**-tRNA complex revealed a greater lifetime lengthening relative to that experienced by **pQ-π_2_** when associated with DNA molecules (τ**_pQ-π2+_**_RNA_/τ**_pQ-π2+_**_DNA_ = 1.6), accounting for the higher emission efficiency (QE**_pQ-π2+_**_RNA_/QE**_pQ-π2+_**_DNA_ = 2.1). Given that selectivity is the result of both greater affinity and higher switch-on response upon the interaction with a specific biological target over the others, these results can anticipate an improved RNA-selectivity for the **pQ-π_2_** relative to either the pyridinium derivative and the ortho isomers, which had already proved to show a certain preference to bind RNA in vitro [36].

Fluorescence microscopy images indeed revealed the cell permeability of the dyes and the selective localization of their fluorescent signal in both the nucleolar region and the perinuclear portion of the cytoplasm. Bright red dots associated with RNA-rich nucleoli can be identified within the nuclei, with a sharper definition in the case of **pQ-π_2_**. The assignment of this emission to the specific interaction with nucleolar RNA was corroborated by RNase and DNase digestion tests, which demonstrated how the degradation of RNA causes the fluorescence signal to lose its brightness and peculiar localization while it remains almost unaltered when DNA is digested. Analogously, confocal microscopy measurements performed on the ortho isomers had previously revealed a similar nucleolar localization but also a diffused fluorescence signal in the entire cellular body, which is an index of poor selectivity [36]. In the case of the para isomers, the background fluorescence in the nucleoplasm is reduced when resorting to **pQ-π_2_**, resulting in an improved contrast when lighting up the nucleoli. Moreover, the red signal outside the nucleus is not randomly diffused but limited to a portion of the cytoplasm recognizable as the mitochondria, as evidenced by colocalization experiments with MitoTracker^TM^ Green, giving Pearson’s coefficients of 0.94 and 0.88 for **pQ-π_2_** and **pPy-π_2_**, respectively. The specific localization in the mitochondria could be again related to the interaction with RNA since mitochondria possess their genomes with their own set of RNAs, whose functionality can be dysregulated under pathological conditions, like in cancer cells [50], and thus might need to be monitored. This behavior is similar to that of the cell-permeant SYTO^TM^ RNASelect^TM^ green fluorescent probe, which is the only commercially available dye for live cell staining of RNA-rich regions associated with nucleoli and mitochondria [25,51]. However, while SYTO^TM^ RNASelect^TM^ exhibits green fluorescence with an emission maximum around 530 nm, the investigated dyes have an uncommonly intense fluorescence in the far-red, centered at 700 nm, which represents a remarkable feature for a fluorescent probe as it allows better tissue penetration and helps to cut off the contribution of endogenous autofluorescence [6].

As for **pQ-π_2_**, in a recently-published paper [37], its bright fluorescence had already been visualized in the mitochondria of HeLa cells through laser confocal microscopy and assigned to the experienced greater viscosity, which restricts the excited-state twisting of the molecule, inhibiting the population of the scarcely emissive TICT state. However, the interaction of the probe with nucleic acids, readily available within the cells, was not taken into consideration. As a matter of fact, when dealing with positively-charged aromatic small molecules, their binding to nucleic acids is destined to happen in a cellular environment and is essential to describe their in vivo behavior. Hence, in agreement with the previous literature, the fluorescence switch-on of **pQ-π_2_** can indeed be assigned to a restriction of molecular motion preventing the probe from reaching the fully-relaxed TICT state, but this new study unraveled how the reason beyond this is its distinct binding with RNA.

These results, together with the moderate antiproliferative effect exhibited by the quinolinium derivative at micromolar concentrations, supposedly following the interference with the cell life cycle and metabolism by specific and stronger interactions with nucleic acids, imply the potential use of this molecule for theranostic applications. In conclusion, this study demonstrated the superior properties of the far-red emitting **pQ-π_2_** fluorophore as an in vitro RNA-selective probe relative to its pyridinium analogs and ortho isomers. The results revealed how small changes in the molecular structure (para vs. ortho; quinolinium vs. pyridinium) could fine-tune the performance of the dye, thus guiding the synthesis of novel compounds.

## 4. Materials and Methods

### 4.1. Synthesis

The molecular structures of the two investigated cations **pPy-π_2_** and **pQ-π_2_** are reported in Scheme 1. Their synthetic procedure is described in the following.

4-((1E,3E)-4-(4-(dimethylamino)phenyl)buta-1,3-dien-1-yl)-1-methylpyridin-1-ium iodide (**pPy-π_2_**): to a solution of (E)-3-(4-(dimethylamino)phenyl)acrylaldehyde (88 mg, 0.5 mmol) and 1,4-dimethylpyridin-1-ium iodide (118 mg, 0.5 mmol) in methanol (15 mL), piperidine was added (4.95 mL, 0.05 mmol). The resulting solution was refluxed for 4 h under a dinitrogen atmosphere in the dark. After cooling, the dark solution was evaporated to a volume of 5 mL and left to stand in the dark. The crystals were collected by filtration and washed with ether. After drying under vacuum at 80 °C, the needles obtained weighed 59 mg. Yield: 0.059 g. 30%, dark purple needles. ^1^H NMR ([D_6_]DMSO): δ = 3.01 (s, 6 H, N(C*H*_3_)_2_), 4.17 (s, 3 H, N^+^C*H*_3_), 6.72 (m, 3 H, C*H*= + Ar*H*), 7.00 (m, 2 H, C*H*=), 7.46 (d, ^3^*J*_H-H_ = 8.5 Hz, 2 H, Ar*H*), 7.79 (m, 1 H, C*H*=), 8.01 (d, ^3^*J*_H-H_ = 7.0 Hz, 2 H, Py*H*), 8.68 (d, ^3^*J*_H-H_ = 7.0 Hz, 2 H, Py*H*); MS (positive ESI): m/z = 265.2 [M − I]^+^. HRMS (positive ESI): calculated for C_18_H_21_N_2_^+^ 265.1699, found 265.1101.

4-((1E,3E)-4-(4-(dimethylamino)phenyl)buta-1,3-dien-1-yl)-1-methylquinolin-1-ium iodide (**pQ-π_2_**): to a solution of (E)-3-(4-(dimethylamino)phenyl)acrylaldehyde (200 mg, 1.14 mmol) and 1,4-dimethylquinolin-1-ium iodide (233 mg, 0.82 mmol) in methanol (10 mL), piperidine was added (7.93 mL, 0.08 mmol). The resulting solution was refluxed for 4 h and stirred at room temperature overnight under a dinitrogen atmosphere in the dark. After cooling, the dark suspension was filtered and washed with ether. After drying under vacuum at 80 °C, the needles obtained weighed 25 mg. Yield: 0.025 g. 7%, black needles. All spectroscopic measurements are identical to those already reported in the literature [37].

### 4.2. Characterization

ESI-MS spectra were recorded on a Thermo Fisher API 2000 mass spectrometer. High-resolution mass spectra were acquired on a Waters^®^ SYNAPT^®^ G2-S/Si mass spectrometer (Waters, Wilmslow, UK). NMR experiments were achieved at 27 °C using a Varian Unity S 500 (499.88 MHz for ^1^H) spectrometer. Tetramethylsilane (TMS) was the internal reference for all NMR experiments.

### 4.3. Materials

Spectral and photophysical measurements were performed in various solvents (Fluka, spectroscopic grade): chloroform (CHCl_3_), dichloromethane (DCM), 1,2-dichloroethane (DCE), 2-propanol (2-PrOH), ethanol (EtOH), methanol (MeOH), water (W), and their mixtures. With the aim being to carry out experiments in aqueous media at known concentrations, the two compounds were first dissolved in dimethyl sulfoxide (DMSO) of spectroscopic grade (Sigma-Aldrich, Saint Louis, MO, USA) to prepare concentrated stock solutions (1 mM) to be later diluted in ETN (1 mM EDTA, 10 mM Tris-HCl, 10 mM NaCl) aqueous buffer solutions, pH 7.4. Ethylenediaminetetraacetic acid (EDTA), tris(hydroxymethyl)aminomethane hydrochloride (Tris-HCl), and NaCl were purchased from Sigma-Aldrich (Saint Louis, MO, USA). Baker’s yeast tRNA was purchased from Roche (Mannheim, Germany), and calf thymus DNA (ct-DNA) from Sigma-Aldrich (Saint Louis, MO, USA); they were used after dissolution in sterile ETN. The ct-DNA was additionally sonicated and filtered through a 0.45 µm filter. The concentration of the polynucleotides’ stock solutions was determined spectrophotometrically by recording the incremental absorbance at 258 nm for tRNA (ε = 6900 M^−1^ cm^−1^) and 260 nm for ct-DNA (ε = 6600 M^−1^ cm^−1^). Dulbecco’s modified Eagle’s medium (DMEM), fetal bovine serum (FBS), Trypsin, and Penicillin/Streptomycin were purchased from Euroclone (Pero, Italy). Dimethyl sulfoxide (DMSO) for biological experiments, Trypan Blue powder, 3-(4,5-dimethylthiazol-2-yl)-2,5-diphenyltetrazolium bromide (MTT), Deoxyribonuclease I (DNase I) from bovine pancreas, and Ribonuclease A (RNase A) from bovine pancreas were purchased from Sigma-Aldrich (Saint Louis, MO, USA) and Becton, Dickinson and Company (Franklin Lakes, NJ, USA). MitoTracker™ Green FM Dye was purchased from Thermo Fisher Scientific Inc. (Waltham, MA, USA). Vectashield^®^ Vibrance™ Antifade Mounting Medium containing 4′,6-diamidino-2-phenylindole (DAPI) was purchased from Vector Laboratories Inc. (Newark, CA, USA).

### 4.4. Photophysical Measurements

Absorption spectra were recorded with a Cary 4E (Varian, Palo Alto, CA, USA) spectrophotometer, choosing a spectral bandpass of 2 nm and using cuvettes with 1 cm path length. Fluorescence emission and excitation spectra were detected by a FluoroMax-4P (HORIBA Scientific, Jobin Yvon, France) spectrofluorimeter and analyzed by the FluorEssence software with appropriate instrumental response correction files. The path length of the cuvette was 1 cm, and the acquisition of the spectra was conducted in right-angle geometry. The spectral bandpass was always set at 2 nm for the excitation monochromator, while in emission, the spectral bandpass ranged from 10 to 20 nm, depending on the system under investigation. The fluorescence quantum yields (Φ_F_, experimental error ± 10%) of dilute solutions (A at λ_exc_ < 0.15) were obtained by exciting each sample at the relative maximum absorption wavelength by employing tetracene in air-equilibrated cyclohexane (Φ_F_ = 0.17) [52]or rhodamine 6G in ethanol (Φ_F_ = 0.94) [53] as reference compounds.

### 4.5. Femtosecond Spectroscopy

The experimental setup for the femtosecond transient absorption (fs-TA) and fluorescence up-conversion (fs-FUC) measurements have been widely described elsewhere [54,55,56]. Briefly, the 400-nm excitation pulses of about 60 fs were generated by an amplified Ti:Sapphire laser system (Spectra Physics, Mountain View, CA, USA). The fs-TA spectrometer (Helios, Ultrafast Systems, Sarasota, FL, USA) is characterized by a time resolution of 150 fs and a spectral resolution of 1.5 nm. Probe pulses are produced in the 450–800 nm range by passing a small fraction of the 800 nm excitation radiation through an optical delay line (time window of 3200 ps) and focusing it onto a 2-mm thick Sapphire crystal to generate a white-light continuum. In the fs-FUC setup (Halcyone, Ultrafast System, Sarasota, FL, USA), the 400-nm pulse excites the sample, whereas the fundamental laser beam acts as the “gate” light. After passing through the delay line, the “gate” reaches the Sapphire crystal, where it combines through the up-conversion process with the fluorescence emitted by the sample with the same time delay. The time resolution is about 300 fs, while the spectra resolution is 1.5 nm. All the ultrafast measurements were carried out under the magic angle condition, stirring the solution in a 2 mm cuvette (0.5 < A < 1.0 at λ_exc_ = 400 nm) during the experiments to avoid the occurrence of photoreactions. Photodegradation was, however, checked by recording the absorption spectra before and after each time-resolved measurement. The experimental data matrixes were first analyzed by using the Surface Xplorer PRO (Ultrafast Systems, Sarasota, FL, USA) software, where it was possible to perform SVD of the 3D matrix to derive the principal components (spectra and kinetics) [57,58]. Successively, the Global Analysis through GloTarAn software was performed to obtain the lifetimes and the Evolution-Associated Spectra (EAS) of the detected transient [59]. When carrying out the measurements of **pPy-π_2_** in the presence of nucleic acids, Target Analysis was performed to consider a parallel decay accounting for the independent deactivation of free dye molecules and their bound form with polynucleotides. The results of the Target Analysis are the Species-Associated Spectra (SAS) of the transients with their lifetime.

### 4.6. Quantum Mechanical Calculations

Quantum mechanical calculations were performed by using the Gaussian 16 package (Wallingford, CT, USA) [60]. DFT with the CAM-B3LYP functional was chosen as the method to optimize the ground state geometry of these small organic push-pull systems and derive their properties [61]. Meanwhile the lowest singlet excited states were investigated through TD-DFT excited-state calculations, again resorting to the CAM-B3LYP functional. Calculations were submitted, setting 6-31g+G(d) as the basis set, including the solvent effect (DCM) according to the conductor-like polarizable continuum model (CPCM) [62].

### 4.7. Spectroscopic Titrations

All spectrophotometric and fluorimetric titrations were conducted in ETN aqueous buffer solutions, pH = 7.4, prepared by diluting concentrated stock solutions of the two dyes (DMSO, c = 1.0 mM) in the buffer, allowing to reach a concentration in the micromolar range (1–2 µM) and keep the final DMSO concentration (v/v) < 1%. In the case of spectrofluorimetric titrations, neutral grey filters were put in the excitation line to dampen the intensity of light and avoid the possible photoisomerization of the investigated compounds subject to repeated irradiation. The excitation wavelength was chosen as the maximum of the absorption spectrum of the free molecule (A_λexc_ < 0.15). Titrations were then performed by adding increasing amounts of nucleic acid stock solutions (c ≈ 2 mM, added volumes in the µL range) to the aqueous solution of the studied dye having a starting volume of 2 mL. The total volume of nucleic acids added at the end of the titration was 1.4 mL. After mixing polynucleotides with the investigated compounds at every addition, spectra were recorded after waiting a standard time of 5 min allowing the equilibrium to be reached in the cuvette. The saturation and establishment of a dominant mode of binding were reached in excess of tRNA or ct-DNA r ≤ 0.005 (r = [compound]/[nucleic acid]). Each absorption spectrum was multiplied for the relative dilution factor, while emission spectra were corrected by taking into account the changes in absorbance at the excitation wavelength after every addition. Fluorescence data were processed employing non-linear fitting to the Scatchard equation (McGhee–von Hippel formalism) [63], giving values of the ratio of n = [bound compound]/[polynucleotide phosphates] in the range of 0.1–0.2. For a straightforward comparison, all K_ass_ values were re-calculated at fixed n = 0.2, allowing satisfactory correlation coefficients (>0.99) to be calculated. The appraisal of fluorescence quantum efficiencies (QE) for the tRNA/compound or ct-DNA/compound complexes was then carried out by comparison of the emission spectra of the free ligand (Area_F,free ligand_), used as internal standard, and that of the bound molecule recorded in excess of nucleic acid at the end of the titration corrected for the fraction of absorbed light (Area_F,complex_), according to the following equation: $QE_{complex}=\left(Area_{F,\;complex}/Area_{F,free\;ligand}\right)\times \Phi_{F,free\;ligand}$.

### 4.8. Cell Cultures

A549 (CCL-185™) human alveolar basal epithelial adenocarcinoma cells and HT-29 (HTB-38™) human colorectal adenocarcinoma cells (ATCC, Manassas, VA, USA) were cultured in a DMEM medium containing 10% (*v*/*v*) heat-inactivated FBS and Penicillin 10,000 U per mL/Streptomycin 10 mg per mL. The cell concentration was monitored by Trypan blue dye staining using an automated cell counter (Invitrogen™ Countess™, Thermo Fisher Scientific, Waltham, MA, USA).

### 4.9. Antiproliferative Assay

The MTT assay was used to study the effect of the **pPy-π_2_** and **pQ-π_2_** compounds on cell proliferation. 2 × 10^3^ A549 and HT-29 cells were seeded in Falcon^®^ 96-well clear flat-bottom microplates (Becton, Dickinson and Company, Franklin Lakes, NJ, USA) with 200 µL of DMEM medium. After 24 h of incubation, the medium was replaced with 198 µL of fresh DMEM, and 2 µL of different dilutions of the **pPy-π_2_** and **pQ-π_2_** compounds stock solution (10 mM in DMSO) were added into each well to reach concentrations ranging from 10 to 0.001 μM in quadruplicate. A quadruplet was kept as control (200 µL of DMEM medium), and another quadruplet was used to take into account the contribution of DMSO (vehicle control 198 µL of DMEM medium + 2 µL of DMSO). After 72 h of incubation in a humidified atmosphere with 5% CO_2_ at 37 °C, 20 µL of a 5 mg/mL MTT dye solution was added to each well to reach a final concentration of 0.5 mg/mL. The cells were then incubated in a humidified atmosphere with 5% CO_2_ at 37 °C for 3 h to allow the formation of formazan crystals, which were subsequently dissolved in 150 µL of DMSO at 37 °C for at least 30 min. After a brief mechanical shaking of the microplates, the optical density at 570 nm was determined using a microplate reader (Beckman Coulter DTX880, Beckman Coulter, Inc., Brea, CA, USA). Cell viability was expressed as the optical density percentage in treated cells compared with vehicle controls, assuming the absorbance of controls was 100% (absorbance of treated wells/absorbance of control wells × 100). All measurements were performed in two independent experiments.

### 4.10. Fluorescence Microscopy

A total of 1500 A549 cells were seeded on round glass coverslips previously sterilized by 30 s of immersion in 70% ethanol, rinsed with sterile phosphate buffer saline (PBS), and placed in a Falcon^®^ 24-well clear flat-bottom multiwell cell culture plates (Becton, Dickinson and Company, Franklin Lakes, NJ, USA). The cells were then incubated for 45 min in a humidified atmosphere with 5% CO_2_ at 37 °C, and subsequently, 500 µL of DMEM medium was gently added to each well. After that, cells were incubated for 24 h under canonical culture conditions (humidified atmosphere with 5% CO_2_ at 37 °C). Then, 2 µL of compound solution diluted in 1000 µL of DMEM at the final concentration of 10 μM was then administered to the cells and incubated for 2 h in a humidified atmosphere with 5% CO_2_ at 37 °C. In the case of the DNase and RNase digestion experiments, stained cells were then rinsed with PBS, fixed in 4% paraformaldehyde for 20 min in the dark, and afterward permeabilized by 0.5% Triton X-100 for 2 min at room temperature. After rinsing again twice with PBS, one-third of the wells were treated with 100 μg mL^−1^ RNase A and one-third with 100 μg mL^−1^ DNase I, while PBS was added to the remaining cells to set up a control experiment. All of the cells were then incubated at 37 °C in 5% CO_2_ for 1 h. After one last washing with PBS, coverslips were mounted onto slides with Vectashield^®^ Vibrance™ Antifade Mounting Medium (Vector Laboratories Inc., Newark, CA, USA). As for the colocalization experiments, cells on round glass coverslips stained with the investigated compounds were then rinsed with PBS, and 1000 µL of MitoTracker™ Green FM Dye in PBS at a final concentration of 200 nM was administered to the cells and incubated for 30 min. After this time, cells were rinsed twice with PBS and fixed in 4% paraformaldehyde for 20 min at room temperature. After washing with PBS, samples were mounted, and nuclei were stained with Vectashield^®^ Vibrance™ Antifade Mounting Medium containing 4′,6-diamidino-2-phenylindole (DAPI) (Vector Laboratories Inc., Newark, CA, USA). Image acquisition was performed by using a fluorescence microscope (Eclipse TE2000-S, Nikon, Tokyo, Japan) equipped with the F-View II FireWire camera (Olympus Soft Imaging Solutions GmbH, Münster, Germany) and using Cell^F^ Imaging Software (Olympus Soft Imaging Solutions GmbH, Münster, Germany). Merged images of the compounds and MitoTracker™ Green FM Dye were analyzed using the ImageJ software (version 1.53t) utilizing the JACoP plugin to calculate Pearson’s and Manders’ Coefficients [49].

## Figures and Tables

**Table 1:** Spectral and photophysical properties of the investigated compounds in solvents of different polarity ($E_{T}^{N}$), absorption maximum (λ_abs_), emission maximum (λ_em_), Stokes shift (Δν), and fluorescence quantum yield (Φ_F_).

		pPy-π_2_^a^				pQ-π_2_			
Solvent	$E_{T}^{N}$	λ_abs_/nm	λ_em_/nm	Δν/cm^−1^	Φ_F_	λ_abs_/nm	λ_em_/nm	Δν/cm^−1^	Φ_F_
DCM	0.321	557	705	3770	0.25	591	679	2200	0.053
DCE	0.346	552	710	4030	0.19	584	687	2600	0.042
2–PrOH/DCM 50:50	0.428	523	705	4900	0.14	569	678	2800	0.032
2–PrOH	0.552	504	707	5700	0.071	552	674	3300	0.026
EtOH	0.654	502	709	5820	0.049	548	678	3500	0.016
MeOH	0.765	492	712	6280	0.027	542	686	3900	0.006
W/EtOH 50:50	0.827	484	710	6580	0.038				
W/EtOH 70:30	0.8962	473	708	7020	0.019	538	684	4000	0.006
W	1	447	709	8270	0.0056	511	688	5000	0.002

^a^ Data regarding **pPy-π_2_** are retrieved from ref. [38].

**Table 2:** Fs-TA results of compounds **pPy-π_2_** and **pQ-π_2_** in DCM, MeOH, and W obtained by Global Analysis.

pPy-π_2_			pQ-π_2_			Assignment
DCM	MeOH	W	DCM	MeOH	W	
τ (ps)			τ (ps)			
0.32	0.59		0.14	0.46		Solv./**LE**
2.7						Solv.
110	2.5	0.50	4.1	1.9	0.39	** ICT **
	17	2.4		4.8	1.4	Solv.
** 840 **	** 230 **	** 73 **	** 82 **	** 15 **	** 5.6 **	** TICT **
rest	rest	rest	rest	rest	rest	

**Table 3:** Association constants (K_ass_) of compounds **pPy-π_2_** and **pQ-π_2_** with tRNA and ct-DNA calculated by processing fluorimetric titrations through the Scatchard equation by fixing n = 0.1.

n Fixed = 0.1	K_ass_	
	pPy-π_2_	pQ-π_2_
**tRNA**	(4 ± 2) × 10^3^	(2.4 ± 0.3) × 10^4^
**ct-DNA**	(3.0 ± 0.2) × 10^4^	(1.8 ± 0.2) × 10^5^

**Table 4:** Fs-TA results of compounds **pPy-π_2_** and **pQ-π_2_** in ETN buffer solution, pH 7.4, alone and in the presence of tRNA and ct-DNA, obtained by Global Analysis or Target Analysis.

pPy-π_2_			pQ-π_2_			Assignment
	+tRNA	+ct-DNA		+tRNA	+ct-DNA	
τ (ps)			τ (ps)			
0.50	0.55	1.3	0.39	0.76	0.50	** ICT **
2.4	3.4	6.0	1.4	8.8	8.7	Solv.
** 73 **	** 88 **	** 82 **	** 5.6 **			** TICT **
	*** 1100 ***	*** 1300 ***		*** 500 ***	*** 320 ***	*** Complex ***
rest			rest		rest	

## Data Availability

Data is contained within the article or Supplementary Material.

## Supplementary Materials

The following supporting information can be downloaded at https://www.mdpi.com/article/10.3390/ijms24054812/s1.

## References

[1] Le P., Ahmed N., Yeo G.W. (2022). Illuminating RNA biology through imaging. Nat. Cell Biol..

[2] Chilka P., Desai N., Datta B. (2019). Small Molecule Fluorescent Probes for G-Quadruplex Visualization as Potential Cancer Theranostic Agents. Molecules.

[3] Sunbul M., Jäschke A. (2018). SRB-2: A promiscuous rainbow aptamer for live-cell RNA imaging. Nucleic Acids Res..

[4] Karlsson H.J., Bergqvist M.H., Lincoln P., Westman G. (2004). Syntheses and DNA-binding studies of a series of unsymmetrical cyanine dyes: Structural influence on the degree of minor groove binding to natural DNA. Bioorganic Med. Chem..

[5] Cesaretti A., Mencaroni L., Bonaccorso C., Botti V., Calzoni E., Carlotti B., Fortuna C.G., Montegiove N., Spalletti A., Elisei F. (2022). Amphiphilicity-Controlled Localization of Red Emitting Bicationic Fluorophores in Tumor Cells Acting as Bio-Probes and Anticancer Drugs. Molecules.

[6] Suseela Y.V., Narayanaswamy N., Pratihar S., Govindaraju T. (2018). Far-red fluorescent probes for canonical and non-canonical nucleic acid structures: Current progress and future implications. Chem. Soc. Rev..

[7] Ramesh J., Arunkumar C., Sujatha S. (2019). Dicationic porphyrins bearing thienyl and pyridinium moieties: Synthesis, characterization, DNA interaction and cancer cell toxicity. Polyhedron.

[8] Čipor I., Kurutos A., Dobrikov G.M., Kamounah F.S., Majhen D., Nestić D., Piantanida I. (2022). Structure-dependent mitochondria or lysosome-targeting styryl fluorophores bearing remarkable Stokes shift. Dye. Pigment..

[9] Ihmels H., Karbasiyoun M., Löhl K., Stremmel C. (2019). Structural flexibility versus rigidity of the aromatic unit of DNA ligands: Binding of aza- and azoniastilbene derivatives to duplex and quadruplex DNA. Org. Biomol. Chem..

[10] Fudickar W., Linker T. (2020). Structural motives controlling the binding affinity of 9,10-bis(methylpyridinium)anthracenes towards DNA. Bioorganic Med. Chem..

[11] Doan P.H., Pitter D.R.G., Kocher A., Wilson J.N., Goodson T.I. (2015). Two-Photon Spectroscopy as a New Sensitive Method for Determining the DNA Binding Mode of Fluorescent Nuclear Dyes. J. Am. Chem. Soc..

[12] Tanious F.A., Veal J.M., Buczak H., Ratmeyer L.S., Wilson W.D. (1992). DAPI (4’,6-diamidino-2-phenylindole) binds differently to DNA and RNA: Minor-groove binding at AT sites and intercalation at AU sites. Biochemistry.

[13] Biver T. (2012). Use of UV-Vis Spectrometry to Gain Information on the Mode of Binding of Small Molecules to DNAs and RNAs. Appl. Spectrosc. Rev..

[14] Roeland Boer D., Canals A., Coll M. (2009). DNA-binding drugs caught in action: The latest 3D pictures of drug-DNA complexes. Dalton Trans..

[15] Wang C., Chi W., Qiao Q., Tan D., Xu Z., Liu X. (2021). Twisted intramolecular charge transfer (TICT) and twists beyond TICT: From mechanisms to rational designs of bright and sensitive fluorophores. Chem. Soc. Rev..

[16] Luo X., Xue B., Feng G., Zhang J., Lin B., Zeng P., Li H., Yi H., Zhang X.-L., Zhu H. (2019). Lighting up the Native Viral RNA Genome with a Fluorogenic Probe for the Live-Cell Visualization of Virus Infection. J. Am. Chem. Soc..

[17] Carlotti B., Cesaretti A., Fortuna C.G., Spalletti A., Elisei F. (2015). Experimental evidence of dual emission in a negatively solvatochromic push–pull pyridinium derivative. Phys. Chem. Chem. Phys..

[18] Cesaretti A., Spalletti A., Elisei F., Foggi P., Germani R., Fortuna C.G., Carlotti B. (2021). The role of twisting in driving excited-state symmetry breaking and enhanced two-photon absorption in quadrupolar cationic pyridinium derivatives. Phys. Chem. Chem. Phys..

[19] Cesaretti A., Carlotti B., Germani R., Spalletti A., Elisei F. (2015). Inclusion of push–pull N -methylpyridinium salts within surfactant hydrogels: Is their excited state intramolecular charge transfer mediated by twisting?. Phys. Chem. Chem. Phys..

[20] Viviano-Posadas A.O., Romero-Mendoza U., Bazany-Rodríguez I.J., Velázquez-Castillo R.V., Martínez-Otero D., Bautista-Renedo J.M., González-Rivas N., Galindo-Murillo R., Salomón-Flores M.K., Dorazco-González A. (2022). Efficient fluorescent recognition of ATP/GTP by a water-soluble bisquinolinium pyridine-2,6-dicarboxamide compound. Crystal structures, spectroscopic studies and interaction mode with DNA. RSC Adv..

[21] Zhu H., Fan J., Du J., Peng X. (2016). Fluorescent probes for sensing and imaging within specific cellular organelles. Acc. Chem. Res..

[22] Ranjan N., Arya D.P. (2016). Linker dependent intercalation of bisbenzimidazole-aminosugars in an RNA duplex; selectivity in RNA vs. DNA binding. Bioorganic Med. Chem. Lett..

[23] Weißenstein A., Vysotsky M.O., Piantanida I., Würthner F. (2020). Naphthalene diimide–amino acid conjugates as novel fluorimetric and CD probes for differentiation between ds-DNA and ds-RNA. Beilstein J. Org. Chem..

[24] Cao C., Wei P., Li R., Zhong Y., Li X., Xue F., Shi Y., Yi T. (2019). Ribosomal RNA-Selective Light-Up Fluorescent Probe for Rapidly Imaging the Nucleolus in Live Cells. ACS Sens..

[25] Yoshino Y., Sato Y., Nishizawa S. (2019). Deep-Red Light-up Signaling of Benzo[c,d]indole–Quinoline Monomethine Cyanine for Imaging of Nucleolar RNA in Living Cells and for Sequence-Selective RNA Analysis. Anal. Chem..

[26] Calzoni E., Argentati C., Cesaretti A., Montegiove N., Tortorella I., Bazzucchi M., Morena F., Martino S., Emiliani C., Jurga S., Barciszewski J. (2021). RNA Modifications in Neurodegenerations. Epitranscriptomics.

[27] Lu Y.-J., Deng Q., Hu D.-P., Wang Z.-Y., Huang B.-H., Du Z.-Y., Fang Y.-X., Wong W.-L., Zhang K., Chow C.-F. (2015). A molecular fluorescent dye for specific staining and imaging of RNA in live cells: A novel ligand integration from classical thiazole orange and styryl compounds. Chem. Commun..

[28] Song G., Sun Y., Liu Y., Wang X., Chen M., Miao F., Zhang W., Yu X., Jin J. (2014). Low molecular weight fluorescent probes with good photostability for imaging RNA-rich nucleolus and RNA in cytoplasm in living cells. Biomaterials.

[29] Zhou B., Liu W., Zhang H., Wu J., Liu S., Xu H., Wang P. (2015). Imaging of nucleolar RNA in living cells using a highly photostable deep-red fluorescent probe. Biosens. Bioelectron..

[30] Higuchi K., Sato Y., Togashi N., Suzuki M., Yoshino Y., Nishizawa S. (2022). Bright and Light-Up Sensing of Benzo[c,d]indole-oxazolopyridine Cyanine Dye for RNA and Its Application to Highly Sensitive Imaging of Nucleolar RNA in Living Cells. ACS Omega.

[31] Sato Y., Igarashi Y., Suzuki M., Higuchi K., Nishizawa S. (2021). Deep-red fluorogenic cyanine dyes carrying an amino group-terminated side chain for improved RNA detection and nucleolar RNA imaging. RSC Adv..

[32] Mazzoli A., Carlotti B., Bonaccorso C., Fortuna C.G., Mazzucato U., Miolo G., Spalletti A. (2011). Photochemistry and DNA-affinity of some pyrimidine-substituted styryl-azinium iodides. Photochem. Photobiol. Sci..

[33] Mazzoli A., Carlotti B., Consiglio G., Fortuna C.G., Miolo G., Spalletti A. (2014). Photobehaviour of methyl-pyridinium and quinolinium iodide derivatives, free and complexed with DNA. A case of bisintercalation. Photochem. Photobiol. Sci..

[34] Mazzoli A., Spalletti A., Carlotti B., Emiliani C., Fortuna C.G., Urbanelli L., Tarpani L., Germani R. (2015). Spectroscopic Investigation of Interactions of New Potential Anticancer Drugs with DNA and Non-Ionic Micelles. J. Phys. Chem. B.

[35] Botti V., Cesaretti A., Ban Ž., Crnolatac I., Consiglio G., Elisei F., Piantanida I. (2019). Fine structural tuning of styryl-based dyes for fluorescence and CD-based sensing of various ds-DNA/RNA sequences. Org. Biomol. Chem..

[36] Botti V., Urbanelli L., Sagini K., Tarpani L., Cesaretti A., Fortuna C.G., Elisei F. (2020). Quaternized styryl-azinium fluorophores as cellular RNA-binders. Photochem. Photobiol. Sci..

[37] Zhang Y., Li Z., Hu W., Liu Z. (2019). A Mitochondrial-Targeting Near-Infrared Fluorescent Probe for Visualizing and Monitoring Viscosity in Live Cells and Tissues. Anal. Chem..

[38] Cesaretti A., Foggi P., Fortuna C.G., Elisei F., Spalletti A., Carlotti B. (2020). Uncovering Structure-Property Relationships in Push-Pull Chromophores: A Promising Route to Large Hyperpolarizability and Two-Photon Absorption. J. Phys. Chem. C.

[39] Reichardt C. (1994). Solvatochromic dyes as solvent polarity indicators. Chem. Rev..

[40] Oudar J.L., Chemla D.S. (1977). Hyperpolarizabilities of the nitroanilines and their relations to the excited state dipole moment. J. Chem. Phys..

[41] Horng M.L., Gardecki J.A., Papazyan A., Maroncelli M. (1995). Subpicosecond Measurements of Polar Solvation Dynamics: Coumarin 153 Revisited. J. Phys. Chem..

[42] Park M., Im D., Rhee Y.H., Joo T. (2014). Coherent and Homogeneous Intramolecular Charge-Transfer Dynamics of 1-tert-Butyl-6-cyano-1,2,3,4-tetrahydroquinoline (NTC6), a Rigid Analogue of DMABN. J. Phys. Chem. A.

[43] Carlotti B., Flamini R., Kikaš I., Mazzucato U., Spalletti A. (2012). Intramolecular charge transfer, solvatochromism and hyperpolarizability of compounds bearing ethenylene or ethynylene bridges. Chem. Phys..

[44] Seki H., Onishi S., Asamura N., Suzuki Y., Kawamata J., Kaneno D., Hadano S., Watanabe S., Niko Y. (2018). Bright and two-photon active red fluorescent dyes that selectively move back and forth between the mitochondria and nucleus upon changing the mitochondrial membrane potential. J. Mater. Chem. B.

[45] Zhuang W., Yang L., Ma B., Kong Q., Li G., Wang Y., Tang B.Z. (2019). Multifunctional Two-Photon AIE Luminogens for Highly Mitochondria-Specific Bioimaging and Efficient Photodynamic Therapy. ACS Appl. Mater. Interfaces.

[46] Zinchuk V., Wu Y., Grossenbacher-Zinchuk O. (2013). Bridging the gap between qualitative and quantitative colocalization results in fluorescence microscopy studies. Sci. Rep..

[47] Zinchuk V., Grossenbacher-Zinchuk O. (2011). Quantitative Colocalization Analysis of Confocal Fluorescence Microscopy Images. Curr. Protoc. Cell Biol..

[48] Giordano A., Romano G. (2008). Cell Cycle Control and Dysregulation Protocols.

[49] Bolte S., Cordelières F.P. (2006). A guided tour into subcellular colocalization analysis in light microscopy. J. Microsc..

[50] Anderson G.R., Wardell S.E., Cakir M., Yip C., Ahn Y., Ali M., Yllanes A.P., Chao C.A., McDonnell D.P., Wood K.C. (2018). Dysregulation of mitochondrial dynamics proteins are a targetable feature of human tumors. Nat. Commun..

[51] Haugland R.P. (2010). Assays for cell viability, proliferation and function. The Handbook, A Guide to Fluorescent Probes and Labeling Technologies.

[52] Birks J.B. (1970). Photophysics of Aromatic Molecules.

[53] Montalti M., Credi A., Prodi L., Gandolfi M.T. (2006). Handbook of Photochemistry.

[54] Mencaroni L., Bonaccorso C., Botti V., Carlotti B., Consiglio G., Elisei F., Fortuna C.G., Spalletti A., Cesaretti A. (2021). Nonlinear optical properties of a new panchromatic series of water-soluble bicationic push-pull fluorophores. Dye. Pigment..

[55] Bonaccorso C., Cesaretti A., Elisei F., Mencaroni L., Spalletti A., Fortuna C.G. (2018). New Styryl Phenanthroline Derivatives as Model D−π−A−π−D Materials for Non-Linear Optics. Chem. Phys. Chem..

[56] Cesaretti A., Carlotti B., Elisei F., Fortuna C.G., Spalletti A. (2018). Photoinduced ICT vs. excited rotamer intercoversion in two quadrupolar polyaromatic N -methylpyridinium cations. Phys. Chem. Chem. Phys..

[57] Golub G.H., Loan C.F.V. (2013). Matrix Computations.

[58] Strang G., Strang G., Strang G., Strang G. (1993). Introduction to Linear Algebra.

[59] Snellenburg J.J., Laptenok S., Seger R., Mullen K.M., Stokkum I.H.M. (2012). van Glotaran: A Java-Based Graphical User Interface for the R Package TIMP. J. Stat. Softw..

[60] Frisch M.J., Trucks G.W., Schlegel H.B., Scuseria G.E., Robb M.A., Cheeseman J.R., Scalmani G., Barone V., Petersson G.A., Nakatsuji H. (2016). Gaussian 16 revision a. 03. 2016.

[61] Becke A.D. (1992). Density-functional thermochemistry. I. The effect of the exchange-only gradient correction. J. Chem. Phys..

[62] Barone V., Cossi M. (1998). Quantum Calculation of Molecular Energies and Energy Gradients in Solution by a Conductor Solvent Model. J. Phys. Chem. A.

[63] McGhee J.D., von Hippel P.H. (1974). Theoretical aspects of DNA-protein interactions: Co-operative and non-co-operative binding of large ligands to a one-dimensional homogeneous lattice. J. Mol. Biol..

