# Supplementary material for: Lighting-Up the Far-Red Fluorescence of RNA-Selective Dyes by Switching from Ortho to Para Position
Source: Int J Mol Sci. 2023 Mar 2;24(5):4812. doi: 10.3390/ijms24054812 (PMC10003335; doi:10.3390/ijms24054812)
Supplement: Supplementary file 1 [file ijms-24-04812-s001.zip › ijms-2151251-supplementary.pdf]

Lighting-Up the Far-Red Fluorescence of RNA-Selective Dyes by Switching from Ortho to Para Position

Alessio Cesaretti,[1,*] Eleonora Calzoni,[1] Nicolò Montegiove,[1] Tommaso Bianconi,[1] Martina Alebardi,[1] Maria Antonietta La Serra,[1†] Giuseppe Consiglio,[2] Cosimo Gianluca Fortuna,[2] Fausto Elisei,[1] Anna Spalletti[1]

[1] Department of Chemistry, Biology and Biotechnology and Center of Excellence on Innovative Nanostructured Materials (CEMIN), University of Perugia, via Elce di Sotto 8, 06123 Perugia (Italy)
[2] Department of Chemical Sciences, University of Catania, Viale Andrea Doria 6, 95125 Catania, Italy
† Current address: Laboratory of Molecular Modeling and Drug Design, Istituto Italiano di Tecnologia, Via Morego 30, 16163 Genoa, Italy
***** Correspondence: alex.cesaretti14@gmail.com

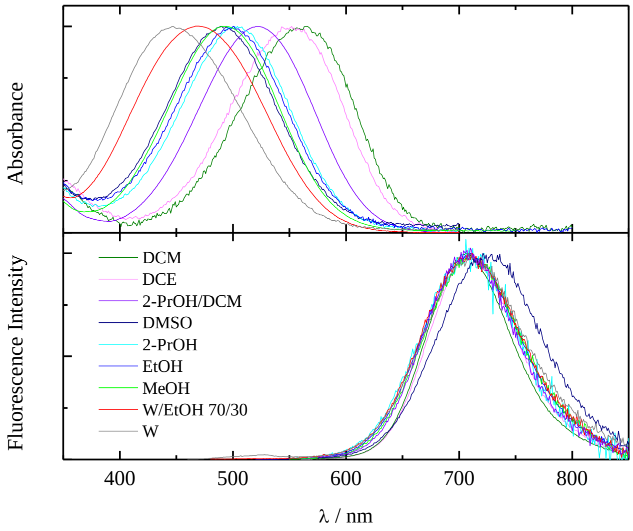

Figure S1. Normalized absorption (upper panel) and emission (lower panel) spectra of **pPy-π₂** in solvents of different polarities. (W – water; EtOH – ethanol; MeOH – methanol; 2-PrOH – 2-propanol; DCE - 1,2-dichloroethane; DCM – dichloromethane). From ref. 38.

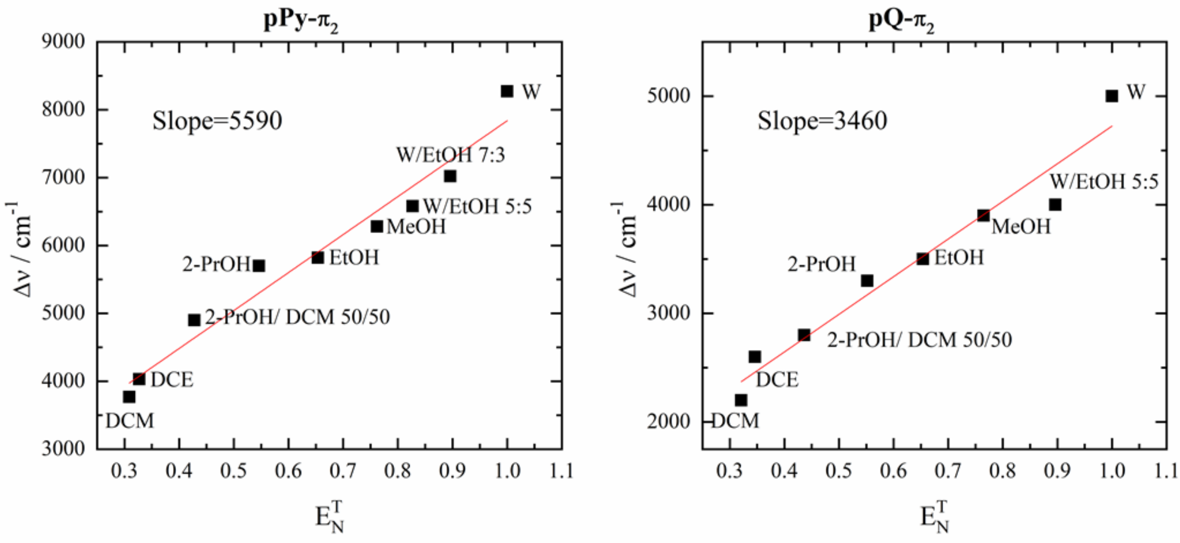

Figure S2. Plot of the Stokes shift as a function of E_T^N parameter for **pPy-*π*$_2$** (**left**) and **pQ-*π*$_2$** (**right**). Data regarding **pPy-*π*$_2$** are retrieved from ref.[38].

Table S1. Predicted and experimental data required for the determination of the dynamic (β_{CT}) and static (β_0) hyperpolarizability coefficients of the investigated compounds in DCM: ground state dipole moment (μ_g); Franck-Condon excited-state dipole moment ($\mu_{e,FC}$); Onsager's cavity ray (a); slope obtained from the fitting of the Stokes shift vs. ETN; dipole moment difference ($\Delta\mu_{exp}$) obtained by applying the solvatochromic method; absorption maximum frequency (υ_{eg}); experimental oscillator strength (f).

Compound	μ_g/D	$\mu_{e,FC}$/D	a/10^{-8} cm	slope	$\Delta\mu_{exp}$/D	υ_{eg}/cm^{-1}	f	β_{CT}/10^{-30} esu^{-1} cm^5	β_0/10^{-30} esu^{-1} cm^5
**pPy-π$_2$**[a]	17.85	1.29	10.1	5590	−13.2	17953	1.068	2840	200
**pQ-π$_2$**	11.83	7.56	10.2	3540	−10.6	16920	0.903	990	160

[a] Data regarding **pPy-*π*$_2$** are retrieved from ref.[38].

Table S2. Absorption wavelengths (λ), oscillator strength (f), and molecular orbitals of **pPy-π₂** in DCM (CPCM) calculated by the CAM-B3LYP/6-31+G(d)//CAM-B3LYP/6-31+G(d) model, together with the experimental absorption maxima. From ref. 38.

Transition	λ_{th}/nm	f	MOs	%	λ_{exp}/nm
$S_0 \rightarrow S_1$	497	1.9337	$\pi_H \rightarrow \pi^*_L$	89	557
$S_0 \rightarrow S_2$	299	0.0203	$\pi_{H-1} \rightarrow \pi^*_L$	65	
$S_0 \rightarrow S_3$	284	0.0466	$\pi_H \rightarrow \pi^*_{L+4}$	45	
$S_0 \rightarrow S_4$	282	0.0014	$\pi_H \rightarrow \pi^*_{L+1}$	76	
$S_0 \rightarrow S_5$	270	0.1947	$\pi_H \rightarrow \pi^*_{L+2}$	44	
$S_0 \rightarrow S_6$	249	0.1624	$\pi_{H-2} \rightarrow \pi^*_L$	70	
$S_0 \rightarrow S_7$	243	0.0405	$\pi_H \rightarrow \pi^*_{L+5}$	78	
$S_0 \rightarrow S_8$	234	0.1502	$\pi_{H-4} \rightarrow \pi^*_L$	81	
$S_0 \rightarrow S_9$	225	0.0001	$\pi_H \rightarrow \pi^*_{L+6}$	74	
$S_0 \rightarrow S_{10}$	222	0.0028	$\pi_H \rightarrow \pi^*_{L+3}$<br>$\pi_H \rightarrow \pi^*_{L+7}$	31<br>35	

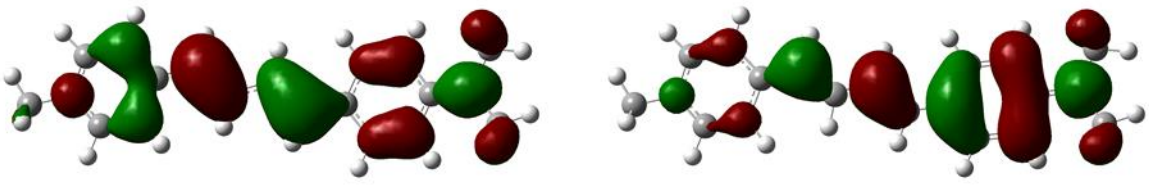

π_{H-1} π_H

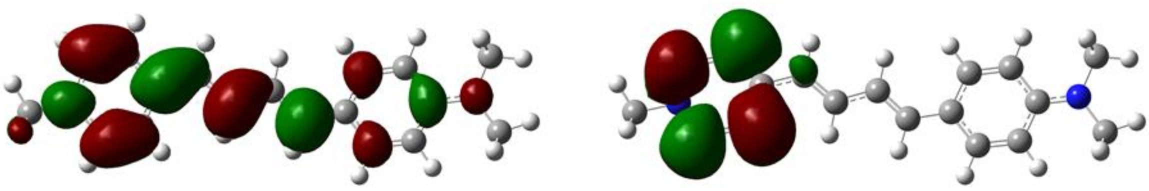

π_L π_{L+1}

Figure S3. Frontier molecular orbitals of **pPy-π₂**.

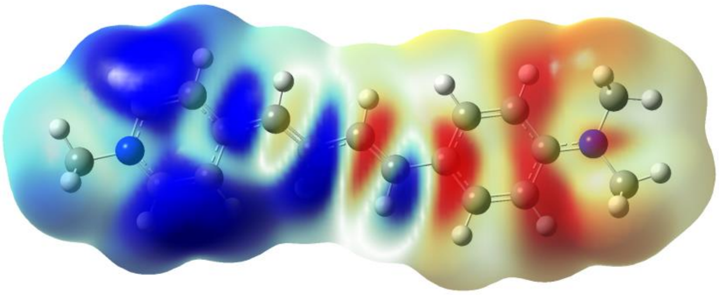

Figure S4. Effect of the $S_0 \rightarrow S_1$ transition on the electron density of **pPy-π₂**; increase and decrease of the electron densities are represented by blue (+0.00005) and red (−0.00005), respectively.

Table S3. Absorption wavelengths (λ), oscillator strength (f), and molecular orbitals of **pQ-π$_2$** in DCM (CPCM) calculated by the CAM-B3LYP/6-31+G(d)//CAM-B3LYP/6-31+G(d) model, together with the experimental absorption maxima.

Transition	λ_{th}/nm	f	MOs	%	λ_{exp}/nm
**$S_0 \rightarrow S_1$**	551	1.9385	$\pi_H \rightarrow \pi^*_L$	88	591
**$S_0 \rightarrow S_2$**	329	0.0001	$\pi_{H-1} \rightarrow \pi^*_L$	72	
**$S_0 \rightarrow S_3$**	298	0.1813	$\pi_H \rightarrow \pi^*_{L+1}$	60	
**$S_0 \rightarrow S_4$**	289	0.1459	$\pi_{H-3} \rightarrow \pi^*_L$	45	
**$S_0 \rightarrow S_5$**	280	0.0378	$\pi_H \rightarrow \pi^*_{L+5}$	34	
**$S_0 \rightarrow S_6$**	279	0.0140	$\pi_{H-4} \rightarrow \pi^*_L$<br>$\pi_{H-3} \rightarrow \pi^*_L$	26<br>28	
**$S_0 \rightarrow S_7$**	265	0.2220	$\pi_H \rightarrow \pi^*_{L+2}$	44	
**$S_0 \rightarrow S_8$**	257	0.2423	$\pi_{H-2} \rightarrow \pi^*_L$	60	
**$S_0 \rightarrow S_9$**	242	0.0394	$\pi_H \rightarrow \pi^*_{L+6}$	76	
**$S_0 \rightarrow S_{10}$**	236	0.0475	$\pi_H \rightarrow \pi^*_{L+3}$<br>$\pi_H \rightarrow \pi^*_{L+5}$	29<br>29	

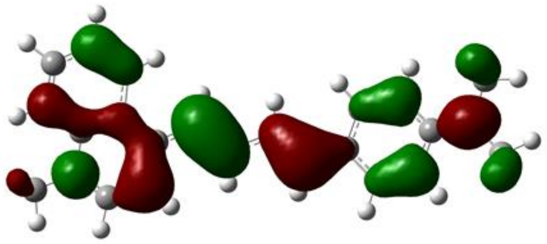

π_{H-1}

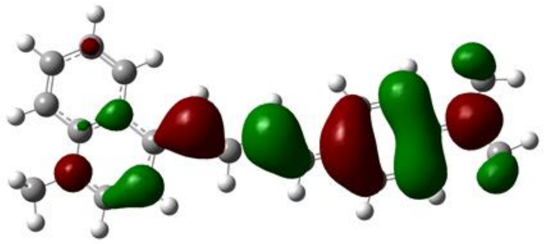

π_H

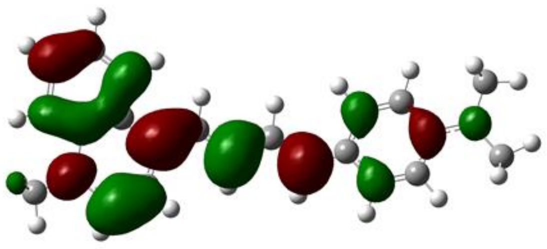

π_L

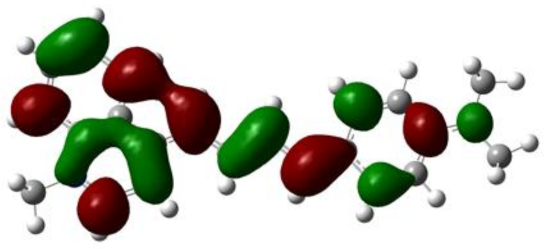

π_{L+1}

Figure S5. Frontier molecular orbitals of **pQ-π$_2$**.

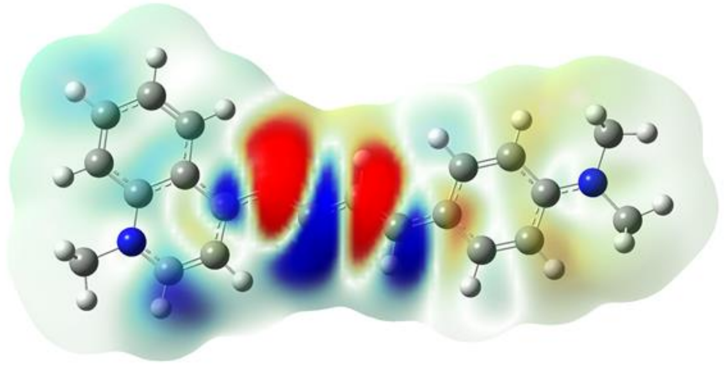

Figure S6. Effect of the $S_0 \rightarrow S_1$ transition on the electron density of **pQ-π₂**; increase and decrease of the electron densities are represented by blue (+0.00005) and red (−0.00005), respectively.

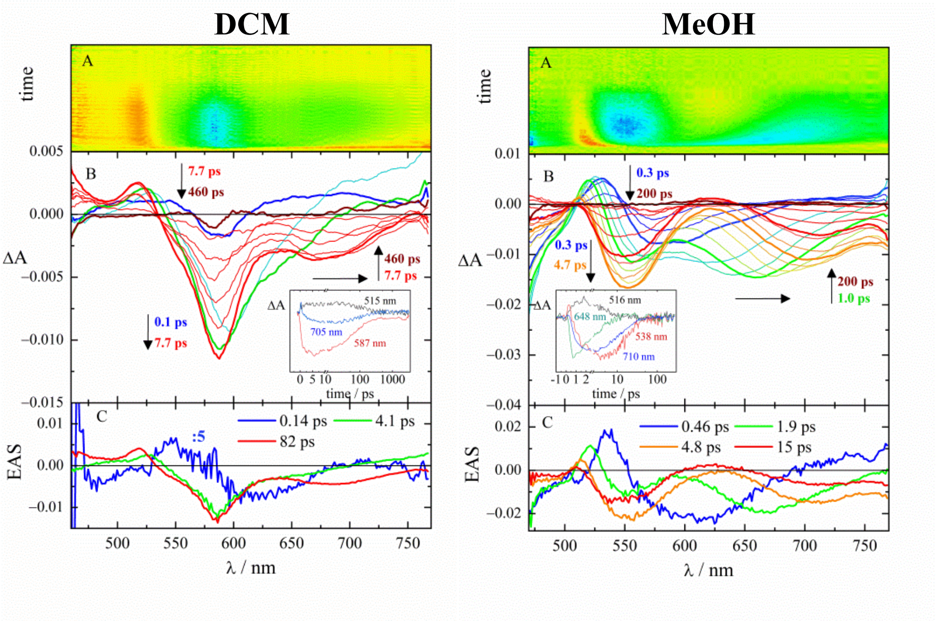

Figure S7. Femtosecond transient absorption (fs-TA) measurements of compound **pQ-π₂** in DCM (**left**) and MeOH (**right**) obtained by pump-probe experiment (λ_{exc} = 400 nm): panel A, experimental 3D matrix reporting color-coded ΔA as a function of wavelength and time (ΔA > 0 yellow-orange, ΔA < 0 blue); panel B, representative spectra at different delay times and representative kinetics (inset) at different wavelengths, together with steady-state absorption (gray-shaded area) and fluorescence (red-shaded area); panel C, EAS (evolution-associated spectra) obtained by Global Analysis.

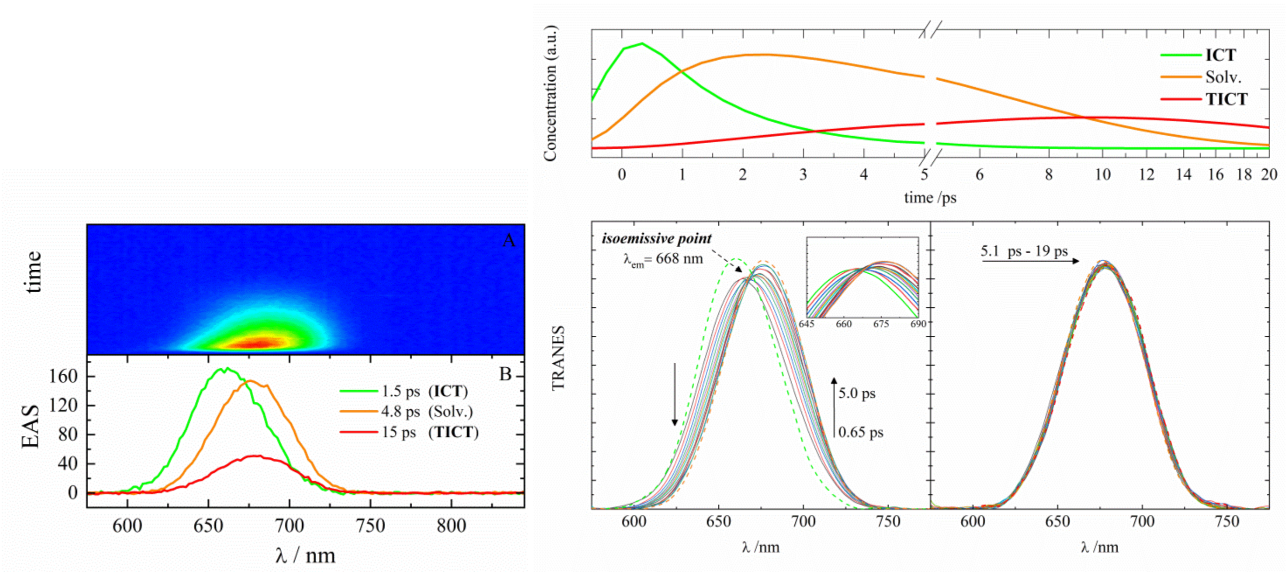

Figure S8. Femtosecond fluorescence up-conversion (fs-FUC) measurement of compound **pQ-π₂** in MeOH obtained by pump-probe experiment (λ_{exc} = 400 nm, **left**): panel A, experimental 3D matrix reporting color-coded ΔA as a function of wavelength and time (ΔA = 0 blue, ΔA > 0 increasing from green to red); panel B, EAS obtained by Global Analysis. Time-resolved area-normalized emission spectra (TRANES) analysis (**right**): upper panel, concentration profiles for the transient species detected by Global Analysis; lower panels, TRANES evolution over time calculated in proper delay time intervals together with the EAS obtained by Global Analysis (dashed lines) reported as limit spectra.

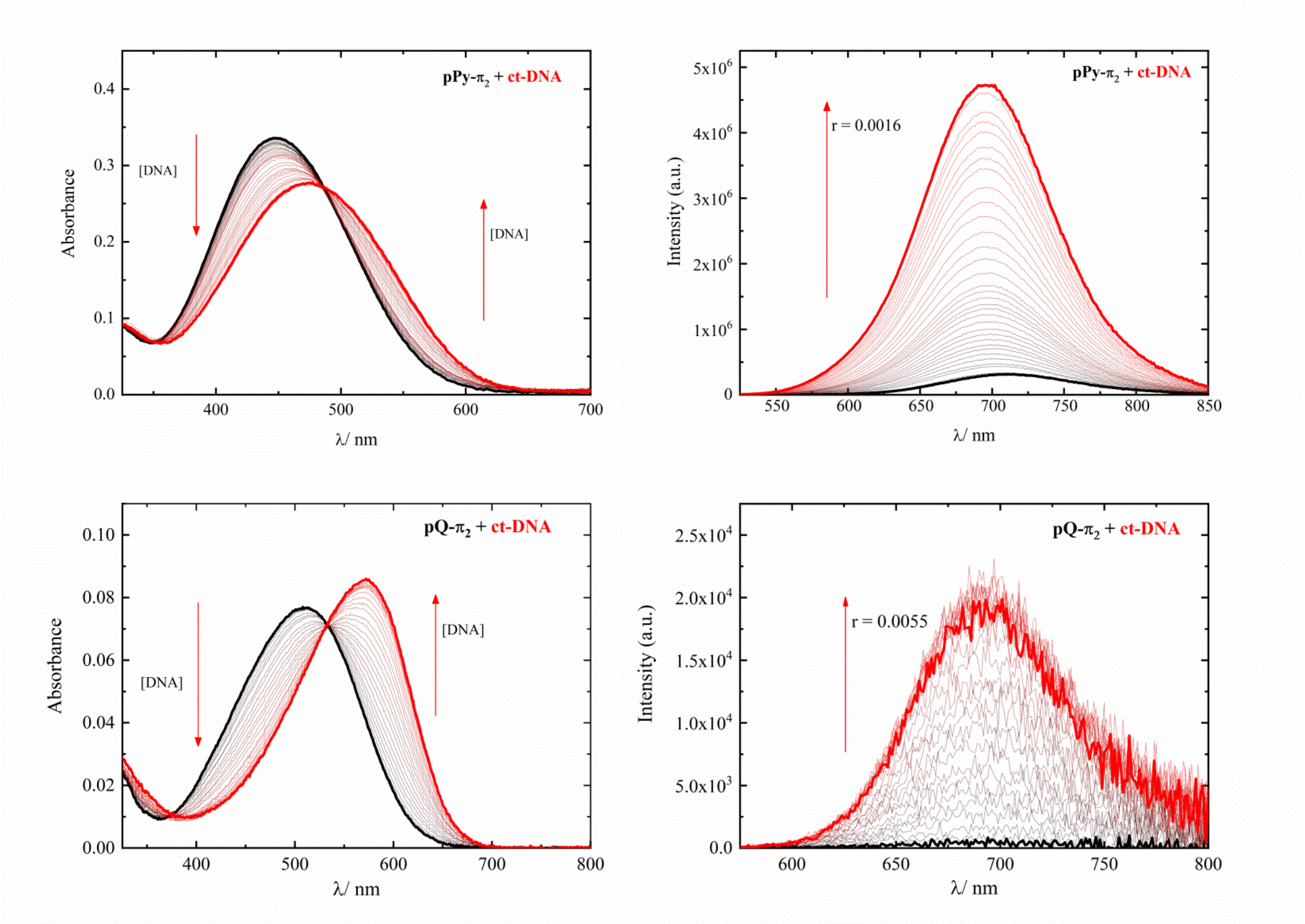

Figure S9. Changes in the absorption (left panels) and fluorescence (right panels) spectra of **pPy-π2** and **pQ-π2** in ETN buffer solution, pH 7.4, upon addition of increasing amounts of ct-DNA, with r = [compound]/[nucleic acid]. [**pPy-π2**] = 1.5 μM, λ_{exc} = 447 nm; [**pQ-π2**] = 1.4 μM, λ_{exc} = 510 nm.

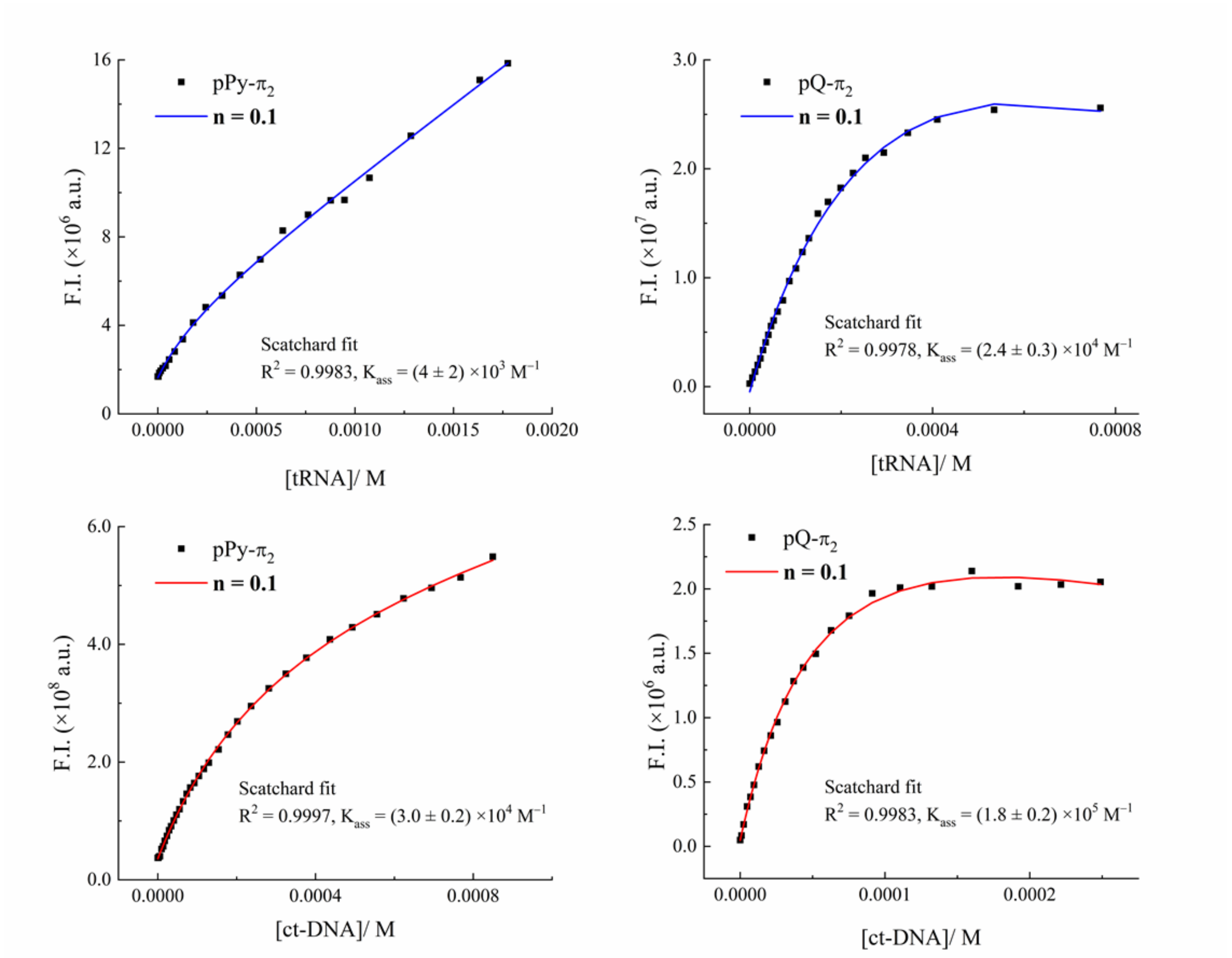

Figure S10. Fluorescence intensities of **pPy-π2** (left) and **pQ-π2** (right) as a function of tRNA (upper) and ct-DNA (lower) concentrations and their fitting according to the Scatchard equation (n = 0.1).

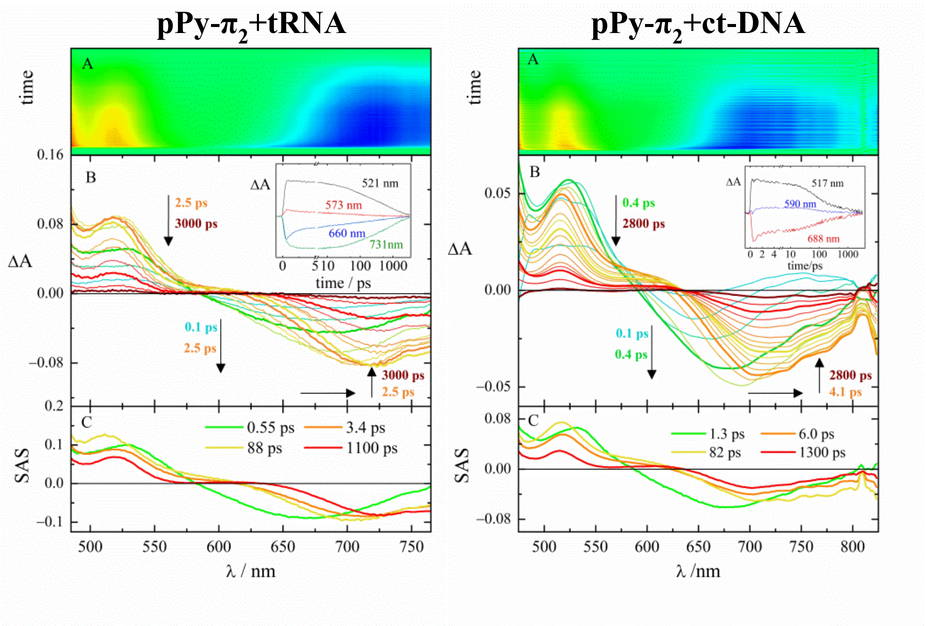

Figure S11. Femtosecond transient absorption (fs-TA) measurements of compound **pPy-π₂** in ETN buffer solution, pH 7.4, in the presence of tRNA (**left**) and ct-DNA (**right**), with r ([compound]/[nucleic acid]) = 0.02, obtained by pump-probe experiment (λ_{exc} = 400 nm): panel A, experimental 3D matrix reporting color-coded ΔA as a function of wavelength and time (ΔA > 0 yellow-orange, ΔA < 0 blue); panel B, representative spectra at different delay times and representative kinetics (inset) at different wavelengths, together with steady-state absorption (gray-shaded area) and fluorescence (red-shaded area); panel C, SAS (species-associated spectra) obtained by Target Analysis.

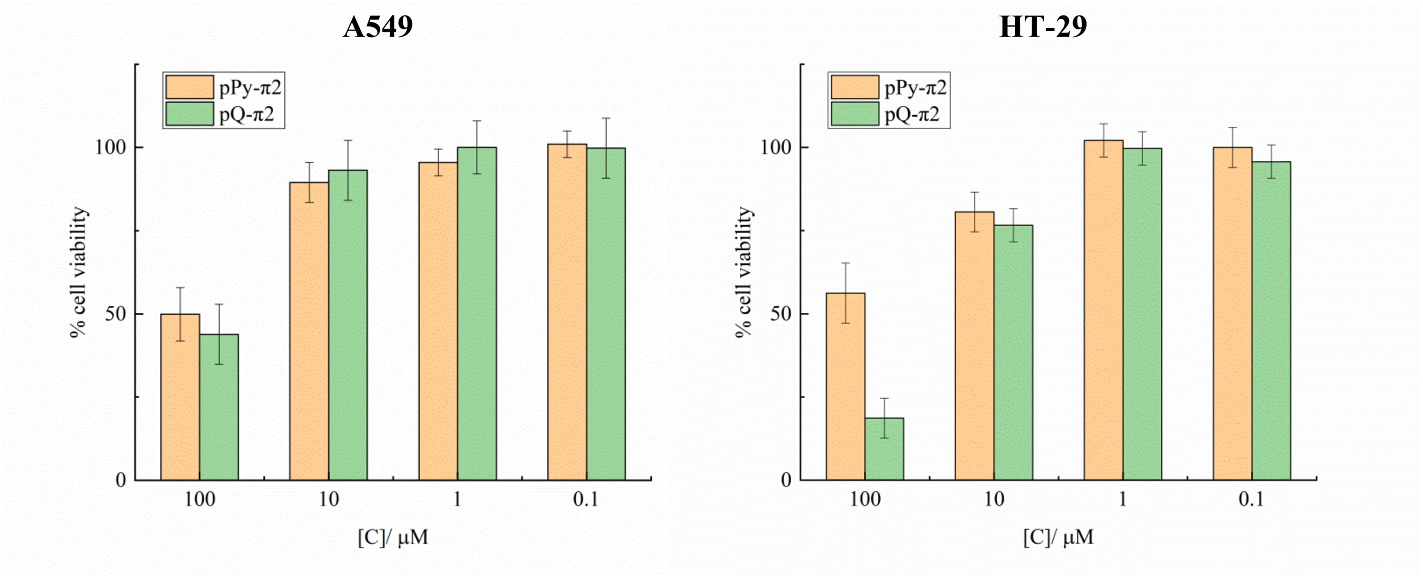

Figure S12. Antiproliferative effect of different concentrations of **pPy-π₂** and **pQ-π₂** on A549 and HT-29 cells expressed as the mean of two independent experiments of four replicas each ± SD. 100% corresponds to control mean values.
